# Field strength–dependent sensitivity of chemical exchange saturation transfer: a methodological comparison of 3 Tesla and 7 Tesla in a clinical cohort

**DOI:** 10.1093/braincomms/fcag248

**Published:** 2026-06-30

**Authors:** Milena Capiglioni, Moritz Simon Fabian, Stefanie Marti, Richard McKinley, Robert Hoepner, Alejandro León Betancourt, Johannes Slotboom, Roland Wiest, Moritz Zaiss, Angelika Mennecke, Piotr Radojewski

**Affiliations:** Institute for Diagnostic and Interventional Neuroradiology, Support Center for Advanced Neuroimaging, University of Bern, 3010 Bern, Switzerland; Translational Imaging Center (TIC), Sitem-insel, 3010 Bern, Switzerland; Department High-Field Magnetic Resonance, Max Planck Institute for Biological Cybernetics, 72076 Tübingen, Germany; Institute of Neuroradiology, University Hospital Erlangen, Friedrich-Alexander-Universität Erlangen-Nürnberg (FAU), 91054 Erlangen, Germany; Department of Neurology, Inselspital, Bern University Hospital and University of Bern, 3010 Bern, Switzerland; Institute for Diagnostic and Interventional Neuroradiology, Support Center for Advanced Neuroimaging, University of Bern, 3010 Bern, Switzerland; Department of Neurology, Inselspital, Bern University Hospital and University of Bern, 3010 Bern, Switzerland; Department of Neurology, Inselspital, Bern University Hospital and University of Bern, 3010 Bern, Switzerland; Institute for Diagnostic and Interventional Neuroradiology, Support Center for Advanced Neuroimaging, University of Bern, 3010 Bern, Switzerland; Translational Imaging Center (TIC), Sitem-insel, 3010 Bern, Switzerland; Institute for Diagnostic and Interventional Neuroradiology, Support Center for Advanced Neuroimaging, University of Bern, 3010 Bern, Switzerland; Translational Imaging Center (TIC), Sitem-insel, 3010 Bern, Switzerland; Institute of Neuroradiology, University Hospital Erlangen, Friedrich-Alexander-Universität Erlangen-Nürnberg (FAU), 91054 Erlangen, Germany; Department of Artificial Intelligence in Biomedical Engineering, Friedrich-Alexander-Universität Erlangen-Nürnberg (FAU), 91052 Erlangen, Germany; Institute of Neuroradiology, University Hospital Erlangen, Friedrich-Alexander-Universität Erlangen-Nürnberg (FAU), 91054 Erlangen, Germany; Institute for Diagnostic and Interventional Neuroradiology, Support Center for Advanced Neuroimaging, University of Bern, 3010 Bern, Switzerland; Translational Imaging Center (TIC), Sitem-insel, 3010 Bern, Switzerland

**Keywords:** CEST, ageing biomarker, ultra-high field, glioma, tumour subtyping

## Abstract

Chemical exchange saturation transfer (CEST) MRI provides insight into tissue metabolism by detecting low-concentration endogenous molecules. While studies at 7 Tesla (7T) have shown enhanced sensitivity and spectral separation, 3 Tesla (3T) remains the clinical standard, and the relative performance of these field strengths in a direct clinical head-to-head comparison remains unclear. This prospective cohort study provides a direct within-subject comparison of multi-pool CEST imaging at 3T and 7T, using age-related tissue changes and glioma molecular subtypes as representative applications of physiological and pathological CEST sensitivity. Forty-three patients (ages 18–76; 18 female) underwent 3T and 7T CEST MRI prior to surgery due to suspected brain tumour; following quality control, 36 datasets were included at 3T and 32 at 7T. CEST amplitudes from amide, amine, aliphatic relayed nuclear Overhauser effect (rNOE) and magnetization transfer pools were quantified in white matter, grey matter, deep grey matter and tumour tissue. A physics-informed conditional autoencoder (PICAE) was applied at 7T to correct B_1_ inhomogeneity. Age effects were tested using linear regression; tumour subtype differences were tested using the Wilcoxon rank-sum test. The significance level was set to *α* = 0.05, and the Holm–Bonferroni procedure was applied to correct for multiple testing. At 7T, amide and rNOE showed robust negative correlations with age in grey matter and deep grey matter, supporting the potential of CEST as an ageing biomarker. Age dependence at 3T was weaker, limited to rNOE (grey matter and deep grey matter) and magnetization transfer (white matter and deep grey matter). In contrast, tumour CEST metrics showed no significant age dependence at either field strength. Trends in relative amide contrast were consistent with prior findings but did not reach statistical significance. Sensitivity to tumour molecular subtype was similar across field strengths. Variability analyses showed that conventional 7T processing introduced higher technical variability than 3T, whereas PICAE substantially reduced variability and improved data quality at 7T. In conclusion, 7T CEST MRI demonstrates higher potential as a non-invasive marker of brain ageing, whereas our simplified pipeline did not yield additional information for tumour subtyping at either field strength. These findings underscore both the enhanced sensitivity and the higher technical demands of 7T, and highlight the importance of advanced correction strategies such as PICAE for robust use of single-transmit 7T CEST.

## Teaser

Capiglioni *et al*. present a direct methodological comparison of multi-pool chemical exchange saturation transfer (CEST) imaging at 3T and 7T in a glioma cohort. 7T shows higher sensitivity to age-related changes in normal-appearing brain tissue but does not improve tumour differentiation under the standard processing pipeline.

## Graphical Abstract

**Graphical Abstract:**
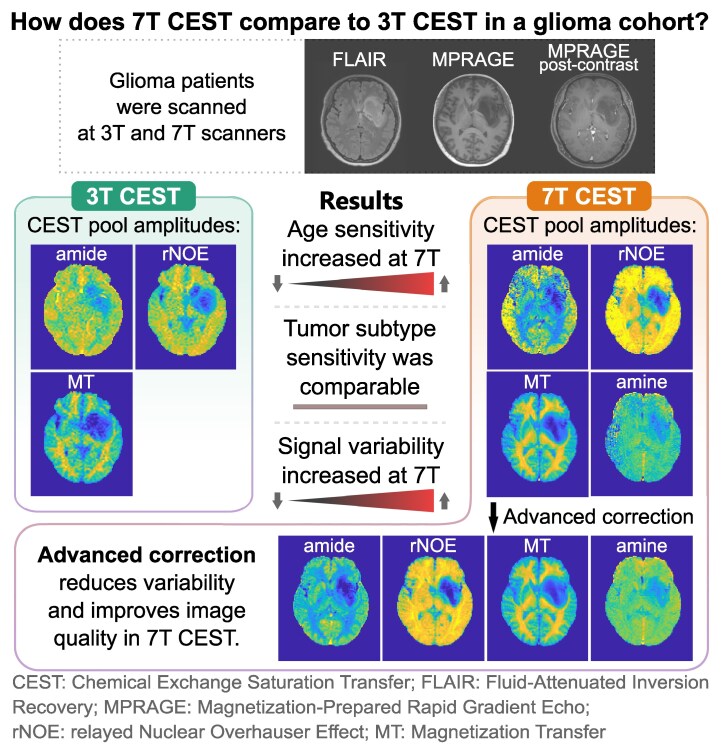

## Introduction

Chemical exchange saturation transfer (CEST) MRI offers insights into tissue metabolism by detecting low-concentration endogenous molecule pools through proton exchange with water.^1,2^ Given its sensitivity to pH, protein content and cellular microenvironment, CEST has been increasingly applied in clinical imaging to probe metabolic alterations associated with disease.^3^

At the clinically established field strength of 3 Tesla (3T), CEST has been applied to a wide range of brain disorders, with the most prominent application being the use of amide proton transfer (APT)-CEST for delineation and histological grade differentiation as well as assessment of progression in brain tumour imaging.^4-7^ pH-weighted APT has also shown promise for delineating ischaemic regions in stroke.^8,9^ Additionally, 3T CEST has been used to investigate neurodegenerative disorders,^10^ including metabolic changes in Alzheimer’s disease,^11,12^ Parkinson’s disease^13^ and multiple sclerosis.^14^

Recent advances in ultra-high field imaging at 7 Tesla (7T) have expanded the potential of CEST by improving chemical shift dispersion, increasing signal-to-noise ratio (SNR) and enhancing saturation efficiency.^15,16^ These advantages come at the cost of increased sensitivity to field inhomogeneities, often requiring more advanced quantification methods than standard Lorentzian fitting.^17^ In recent years, 7T CEST has been increasingly translated into clinical research, with some applications emerging in brain tumour imaging^18^ and multiple sclerosis.^15^ In glioma patients, increased APT- and downfield nuclear Overhauser effect (NOE)-suppressed APT signals with regional variability have been reported,^19^ and the guanidinium CEST pool has shown distinct contrast between enhancing tumour, non-enhancing tumour and contralateral normal WM.^20^ Furthermore, 7T NOE-mediated CEST reveals peritumoural and intratumoural signal alterations not visible on contrast-enhanced T1 or T2-weighted imaging,^21^ while combined 7T CEST/MRS has been proposed as a non-radioactive approach for delineating glioma infiltration.^22^ Together, these studies suggest that 7T CEST captures metabolic heterogeneity in gliomas. Beyond disease, a recent study showed that amide and aliphatic protons (relayed NOE [rNOE]) signals at 7T vary significantly with age in healthy populations,^23^ suggesting potential value as a non-invasive marker for ageing-related processes.

Direct comparisons of 3T and 7T CEST have so far been mainly limited to phantom experiments^24,25^ and animal models,^24^ which consistently show stronger and more specific CEST contrasts at 7T. In practice, however, 3T remains the clinical standard as 7T scanners are not widely approved for routine clinical use, and there is a lack of *in vivo* studies directly comparing 3T and 7T acquisitions in the human brain within the same clinical cohort. Conducting such *in vivo* methodological comparisons is essential to guide the translation of 7T CEST into clinical practice and to determine the relative sensitivity and limitations of each field strength.

In this prospective study, we address this gap by directly comparing CEST acquisitions at 3T and 7T in the same cohort of glioma patients. Our primary aim is to determine whether 7T provides additional or more specific information than 3T, and how sensitivity to age and tumour subtype differs between field strengths, rather than investigating the biological mechanisms of glioma or ageing. Understanding these methodological differences is key for the subsequent successful translation of CEST into clinical practice and for evaluating its utility for metabolic tissue characterization across field strengths.

## Materials and methods

**Figure 1:**
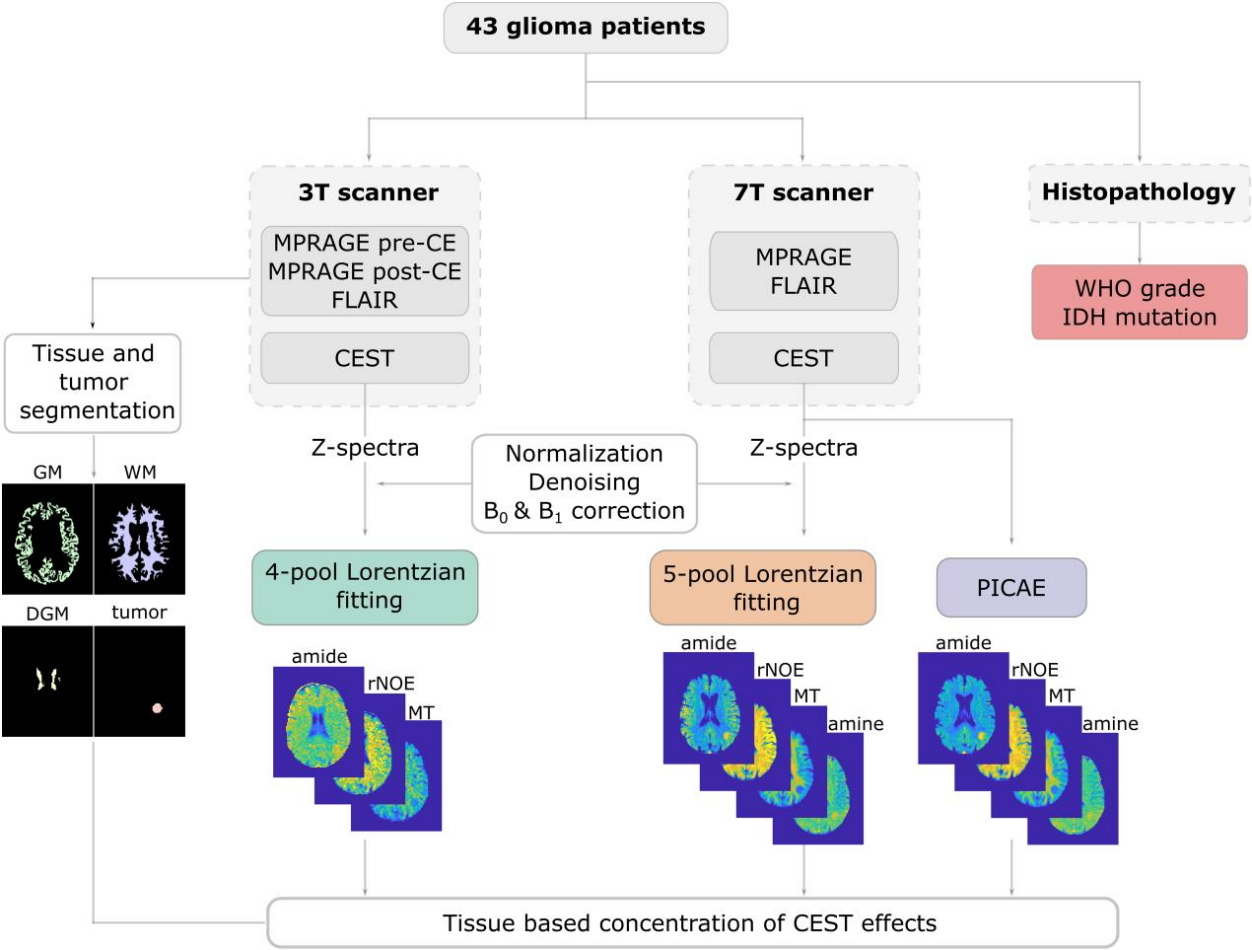
**Overview of MRI processing and analysis pipeline.** Imaging and analysis workflow in 43 glioma patients scanned at 3 Tesla (3T) and 7 Tesla (7T). Structural imaging included magnetization-prepared rapid gradient-echo (MPRAGE; pre- and post-contrast enhancement [CE]) and fluid-attenuated inversion recovery (FLAIR), in addition to chemical exchange saturation transfer (CEST). Segmentation identified tumour, white matter (WM), grey matter (GM) and deep grey matter (DGM) regions. Following normalization, denoising and B_0_/B_1_ correction, Lorentzian fitting yielded amide, relayed nuclear Overhauser effect (rNOE), magnetization transfer (MT) and amine contributions (five-pool model at 7T). Physics informed conditional autoencoder (PICAE) was additionally used to quantify 7T CEST. Histopathology provided World Health Organization (WHO) grading and isocitrate dehydrogenase (IDH) mutation status.

Figure 1 outlines the overall acquisition and post-processing pipeline, explained in detail in the following subsections.

### Patients

We enrolled patients with suspected brain tumours as part of a prospective comparative study on preoperative tumour characterization using 3T and 7T MRI. Inclusion criteria were suspected brain tumour on recent MRI, no contraindications for 7T imaging and no history of neurodegenerative disease. Written histopathological and molecular reports (using the 2021 CNS WHO classification) were assessed to obtain the final integrative diagnosis, including CNS WHO Grade and the presence of isocitrate dehydrogenase (IDH) mutation. The study was approved by the local ethics committee, and all participants provided written informed consent.

### MRI acquisition protocol

All patients underwent scanning on both a 7T scanner (Terra, Siemens Healthcare, Erlangen, Germany) with a 32Rx/1Tx head coil and a 3T scanner (Prisma, Siemens Healthcare, Germany) with a 64Rx/1Tx head coil on the same day. The 7T scan was taken first, directly followed by the 3T acquisition.

### 7T acquisition protocol

CEST imaging at 7T followed a previously described protocol optimized for reproducible multi-pool evaluation.^23^ The pre-saturation module applied two average B_1_ pulse amplitudes (0.72 and 1.00 µT) and sampled 54 non-equidistant frequency offsets between −100 and 100 ppm, with 39 centred between −5 and 5 ppm, and two reference scans at ±300 ppm. Image readout used a 3D snapshot gradient echo (GRE) sequence with centric *k*-space ordering.^26^ B_1_ mapping was performed using a Turbo-FLASH sequence. High-resolution anatomical scans included T2-weighted fluid-attenuated inversion recovery (FLAIR) imaging in all subjects. In 25 participants, MP2RAGE images were acquired for quantitative T1 mapping, while the remaining 18 underwent MPRAGE imaging for anatomical reference.

### 3T acquisition protocol

The CEST 3T protocol also used a pulsed saturation scheme with two average B_1_ amplitudes (0.6 and 0.9 µT), sampling 55 non-equidistant frequency offsets from −100 to 100 ppm, with 45 offsets between −10 and 10 ppm, and a reference scan at −300 Hz, as described previously.^27^ Readout also used a 3D snapshot GRE sequence with centric *k*-space ordering.^26^ For B_0_ and B_1_ mapping, we used the WASABI sequence.^28^ All 3T CEST acquisitions occurred prior to gadolinium administration. Structural imaging included FLAIR and pre- and post-contrast MPRAGE sequences for lesion detection and tissue segmentation.

A complete list of acquisition parameters for both field strengths is provided in Supplementary Material 1. Additionally, because the acquisition of the 3T anatomical scans was determined by clinical needs at the time, the specific scans acquired for the different subject groups are detailed in Supplementary Table 1.

### Post-processing

#### Anatomical segmentation

To classify anatomical regions, we segmented pre-contrast MPRAGE images with DL + DiReCT,^29^ using the Destrieux atlas^30^ to delineate white matter (WM), grey matter (GM), and deep grey matter (DGM). The DGM regions included the hippocampus, thalamus, caudate, putamen, pallidum, brainstem, amygdala and ventral diencephalon. An in-house developed deep learning algorithm generated tumour masks and associated uncertainty maps using 3T FLAIR and pre-/post-contrast MPRAGE images.^31^ The tumour masks delineated the whole lesion without sub-compartmental differentiation (e.g. cystic, necrotic, or oedematous regions). This choice ensured consistent and reproducible segmentation across a heterogeneous cohort and allowed comparable masks to be applied for the 3T–7T comparison. Segmentation masks from 3T were non-linearly registered to the 7T space using SPM.

#### CEST quantification

At both field strengths, we applied multi-Lorentzian fitting to motion-corrected, normalized, denoised and B_0_/B_1_-corrected Z-spectra to extract CEST metrics.^23^ We used a four-pool model at 3T (water, amide, rNOE and magnetization transfer [MT])^27,32^ and a five-pool model at 7T (water, guanidine, amide, rNOE and MT).^23,33^ The fitting procedure returns amplitude, width and centre for each Lorentzian. To account for the increased impact of B_1_ inhomogeneity at 7T, we further quantified metabolites using the physics-informed conditional autoencoder (PICAE) pipeline.^17^

#### Quality control and tissue mask definition

A neuroradiologist (13 years of experience) visually reviewed the automatic tumour segmentations for all patients. We excluded patients if the tumour volume exceeded 25% of the combined WM and GM volume, as such tumours compromise DL + DiReCT performance. We also excluded patients if more than 20% of the data showed a B_0_ shift greater than 0.2 ppm, if B_1_ deviations exceeded 50% from the nominal value in more than 20% of the data, or if severe artefacts were present in the CEST outputs.

We defined normally appearing tissue masks by subtracting the tumour and its uncertainty map (threshold at 10%) from the WM, GM, and DGM maps generated by DL + DiReCT. The 10% threshold was visually selected to fully exclude tumour tissue, acknowledging that this may exclude small parts of normal-appearing tissue.

### Statistical analysis

Following pre-processing and model fitting, Lorentzian amplitudes for each CEST pool were extracted. For each pool, tissue and field strength combination, we fitted a linear regression of the mean CEST amplitude on age and sex. For tumour tissue, a histologically defined tumour type (according to the WHO classification)^34^ was added as an additional covariate. The statistical significance of the contribution of age for each pool, tissue and field strength combination was assessed under the Holm–Bonferroni correction for multiple testing over the respective family of tests at *α* = 0.05. We report the coefficients of age, their 95% confidence intervals and the corresponding uncorrected *P*-values, and indicate the significance after correction.

We evaluated the correlation of CEST effects with histopathological findings in two ways. First, the mean amplitude of each CEST pool was calculated within the tumour mask (plus 10% uncertainty margin) for all included subjects. We assessed differences across WHO classifications using a Kruskal–Wallis^35^ test to account for non-normal data distribution and unequal group sizes. Differences by IDH mutation status were assessed using the Wilcoxon rank-sum^36^ test to account for the non-normal distribution of the data, which was assessed using the Shapiro–Wilk test. We report uncorrected *P*-values and indicate the significance after Holm-Bonferroni correction at *α* = 0.05.

Second, because tumours were located in different brain tissues and regions, we calculated the relative difference in CEST effects between tumour and contralateral healthy tissue as: 100 × (CEST_healthy − CEST_tumor)/CEST_healthy. Since many patients had tumours extending beyond one hemisphere, only those with tumours fully within one hemisphere and allowing for the definition of contralateral normally appearing reference region were included in this analysis (see Supplementary Fig. 1, for examples). For these cases, a reduced tumour mask (excluding boundaries and uncertainty) was mirrored into the contralateral hemisphere. Due to the limited sample size, this analysis was restricted to IDH mutation status and used the same statistical test as described above.

Finally, to assess the reliability of the CEST effect estimates at different field strengths and enable comparison with previous studies, we computed two coefficients of variation (CoVs). The first, CoV model(*p*, *t*), was defined as CoV model(*p*, *t*) = std(y_val(*p*, *t*) −  y_fit(*p*, t))/mean(y_fit(*p*, *t*)), where y_val(*p*, *t*) and y_fit(*p*, *t*) are the observed and model-fitted CEST values, respectively, for pool *p* in tissue *t*. This metric reflects the proportion of variance in CEST values not explained by the age-dependent model, capturing inter-subject variability within each tissue and pool. The second metric, the individual-level coefficient of variation (coV), was defined as CoV(*p*, *t*) = 100 × mean (std (*p*, *t*)/mean (*p*, *t*)), where *p* represents the voxel-wise CEST values within tissue *t* for each subject. This measure quantifies intra-subject variability of CEST signals across the tissue mask.

## Results

A total of 43 patients with suspected brain tumours (mean age 43 ± 15 years; range 18–76 years) were initially enrolled for preoperative tumour characterization. Seven patients were excluded from the analysis: four due to very large tumour volume and three due to incomplete or erroneous acquisition. At 3T, no additional subjects were excluded, resulting in a final sample of 36 patients. At 7T, four additional patients were excluded: three due to severe B_1_ inhomogeneities and one due to incomplete acquisition, leaving 32 patients available for 7T analysis. Examples of CEST maps of excluded subjects (Supplementary Fig. 2), as well as the age distribution of the subjects included at each field strength (Supplementary Fig. 3) can be found in Supplementary Material 3.

**Figure 2:**
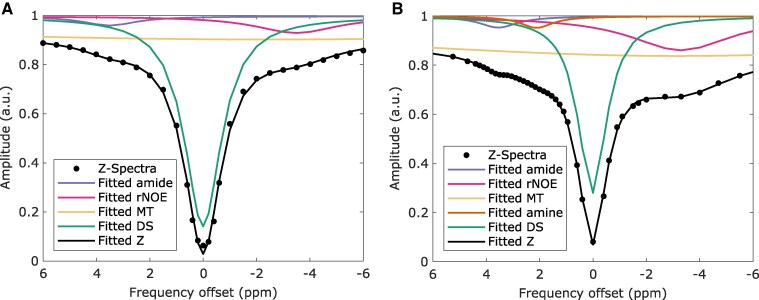
**Representative Z-spectra and multi-pool Lorentzian fits at 3T and 7T.** Z-spectra from a single patient (*N* = 1) are shown for (**A**) 3T and (**B**) 7T. The spectra were averaged across all normally appearing tissue voxels within a slice. Amide, rNOE, MT and direct saturation (DS) model fits are shown at both field strengths, with the additional amine component at 7T. The fitted Z-spectrum (solid black line) reflects the sum of all components.

Figure 2 shows representative Z-spectra and corresponding multi-Lorentzian fits from a single patient at both 3T and 7T. Spectra were averaged across all normally appearing tissue voxels (excluding tumour) within the slice where the tumour appeared largest at each field strength, to select anatomically comparable slices. Lorentzian pools corresponding to amide, rNOE, MT and direct saturation (DS) are shown at 3T, along with amine at 7T.

**Figure 3:**
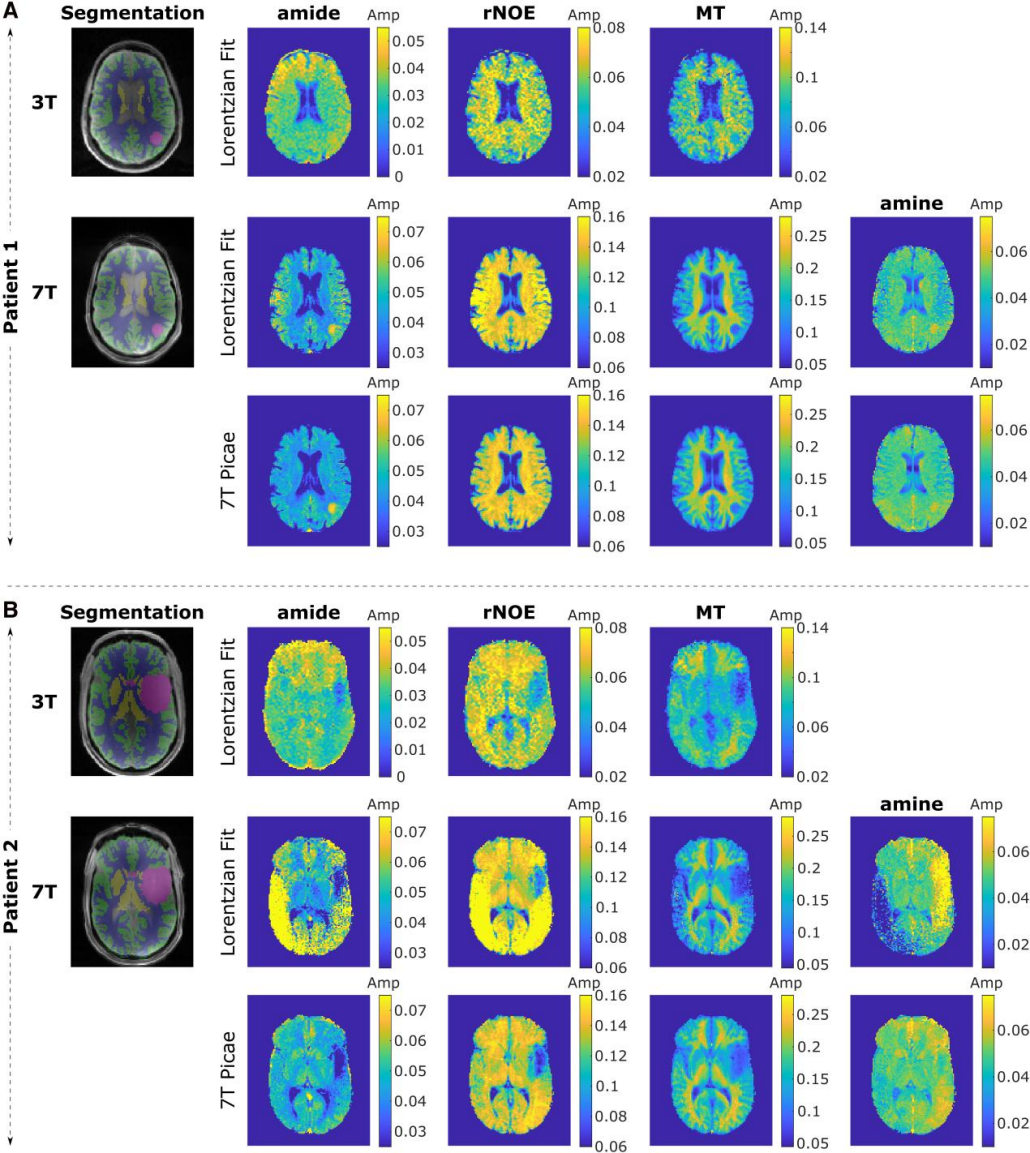
**Comparison of segmentation and CEST metabolite maps at 3T and 7T for two glioma patients.** (**A**) A 71-year-old female patient and (**B**) a 50-year-old male patient. For each acquisition, the segmented tumour overlay, and fitted Lorentzian amplitude maps for four CEST pools (amide, aliphatic rNOE, MT and amine) are shown. At 7T, maps are displayed for both Lorentzian fitting and physics-informed conditional autoencoder (PICAE) quantification. The colour map and corresponding colour scale represent the amplitude of the Lorentzian fitting for each CEST pool in dimensionless units.

Figure 3 illustrates segmentation results and fitted Lorentzian amplitudes for two representative patients, highlighting the improved SNR and CEST contrast at 7T compared to 3T. The corresponding B_0_ and B_1_ field maps for each patient and field strength are provided in Supplementary Fig. 4. Visual inspection of the PICAE-corrected results indicates improved artefact suppression in comparison with the standard multi-pool fitting, particularly in regions affected by B_1_ inhomogeneity. Additional examples across a range of tumour grades and sizes, together with high-resolution anatomical images, are provided in Supplementary Material 5 (Supplementary Figs 5–8).

### Age dependence of CEST pools

**Figure 4:**
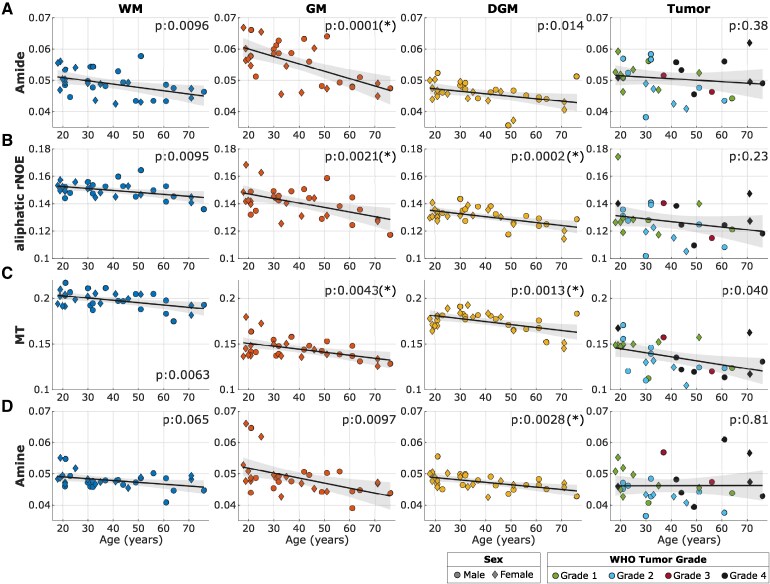
**Age dependence of CEST pool amplitudes across brain tissue types at 7T.** CEST pool amplitudes for (**A**) amide, (**B**) aliphatic rNOE (**C**) MT and (**D**) amine are plotted against age across four tissue types: white matter (WM), GM, deep grey matter (DGM) and tumour tissue. Each point represents the mean value across the corresponding tissue in one patient (*N* = 32). Each plot shows the regression line of the CEST amplitude on age with 95% confidence intervals displayed as shaded grey areas. Dots indicate male patients; diamonds indicate female patients; colours indicate WHO tumour grade. Linear regressions were performed with *α* = 0.05, and uncorrected *P*-values for the age effect are shown with up to two significant digits. Significant age effects after Holm–Bonferroni correction are marked with an asterisk. Full statistical results are provided in Supplementary Table 2.

Figure 4 shows the linear regressions between each CEST pool and age across different tissue types at 7T. Detailed regression outcomes, when considering sex and tumour covariates, are provided in Supplementary Table 2. Statistically significant negative correlations were observed in GM for the amide, aliphatic rNOE and MT pools. DGM showed significant correlation in rNOE, MT and amine pools. WM and tumour tissues showed no statistically significant associations with age.

**Figure 5:**
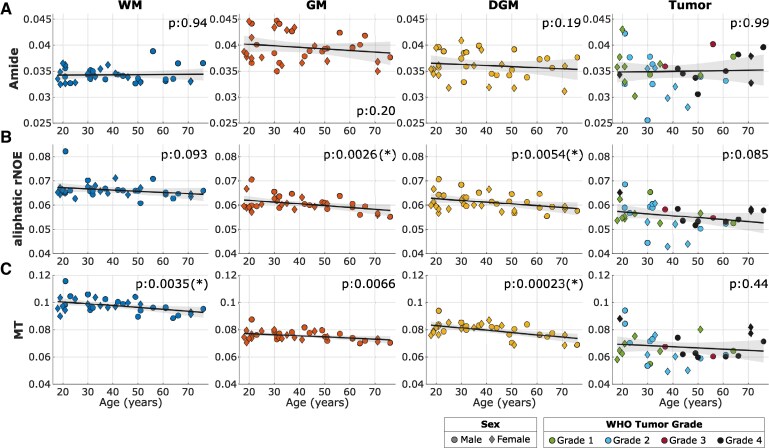
**Age dependence of CEST pool amplitudes across brain tissue types at 3T.** CEST pool amplitudes for (**A**) amide (**B**) aliphatic rNOE, and (**C**) MT plotted against age across four tissue types: white matter (WM), grey matter (GM), deep grey matter (DGM) and tumour tissue. Each point represents the mean value across the corresponding tissue in one patient (*N* = 36). Each plot shows the regression line of the CEST amplitude on age with 95% confidence intervals displayed as shaded areas. Dots indicate male patients; diamonds indicate female patients; colours indicate WHO tumour grade. Linear regressions were performed with *α* = 0.05, and uncorrected *P*-values for the age effect are shown with up to two significant digits. Significant age effects after Holm–Bonferroni correction are marked with an asterisk. Full statistical results are provided in Supplementary Table 3.

At 3T (Fig. 5), weaker age-related effects were observed. MT showed a significant correlation in WM and DGM, while aliphatic rNOE showed significant correlation with age in GM and DGM. No significant age effects were detected in tumour tissue. Regression coefficients are given in Supplementary Table 3.

### IDH mutation and tumour grading

Histopathological and molecular analysis identified IDH mutations in 20 patients, wildtype in 11, and unknown status in 12 (no surgery). Tumour grades were distributed as follows: grade 1 (*N* = 10), grade 2 (*N* = 16), grade 3 (*N* = 3) and grade 4 (*N* = 14).

**Figure 6:**
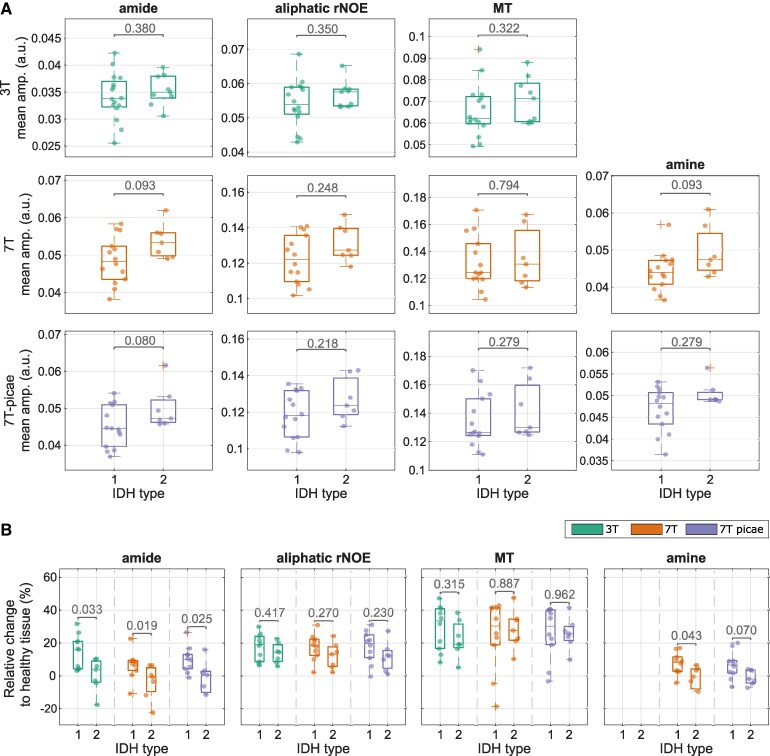
**CEST pool amplitudes across IDH mutation status.** (**A**) Boxplots show the mean tumour amplitudes for each CEST pool (amide, aliphatic rNOE, MT and amine) across IDH mutation status (1 = IDH-mutant, 2 = IDH-wildtype) at 3T (N_1_ = 16, N_2_ = 9), 7T (N_1_ = 14, N_2_ = 7), and 7T PICAE (N_1_ = 14, N_2_ = 7), individual data points representing the mean tumour signal for each patient are overlaid. (**B**) Per cent relative changes in tumour pool amplitudes compared to contralateral normally appearing tissue in the same subject at 3T, 7T and 7T PICAE (N_1_ = 10, N_2_ = 7 for all); individual data points representing the per cent relative change for each patient are overlaid. *P*-values from two-sided Wilcoxon rank-sum tests are indicated above each comparison.

Figure 6A compares tumour CEST amplitudes across IDH mutation status at 3T and 7T with standard Lorentzian fitting, and at 7T with PICAE. Uncorrected *P*-values are shown in each case. No statistically significant differences were observed across any CEST pool after Holm-Bonferroni correction. Supplementary Fig. 9 shows a similar lack of association between WHO tumour grade (grades 1–4) and CEST amplitudes.

Figure 6B presents the per cent difference in tumour CEST amplitude relative to contralateral normal-appearing tissue, with uncorrected *P*-values shown in each case. Following Holm–Bonferroni correction, none of the observed differences reached statistical significance. Nonetheless, the amide signal in IDH-mutant tumours consistently exhibited higher values compared to contralateral tissue at 3T (uncorrected *P* = 0.03), 7T (uncorrected *P* = 0.02) and 7T PICAE (uncorrected *P* = 0.03), indicating a reproducible trend suggestive of a potential biological effect. The amine signal, which was only detectable at 7T, showed nominal significance prior to correction (uncorrected *P* = 0.04) and a similar tendency in the 7T PICAE dataset (uncorrected *P* = 0.07). No appreciable differences were found in the aliphatic rNOE or MT pools.

While Figure 6B suggests potential trends in CEST signal differences across tumour subtypes, the lack of statistically significant group-level differences following correction may in part reflect underlying biological variability. Figure 3 illustrates such variability with an example: two patients with the same diagnosis (glioblastoma, WHO grade 4, IDH-wildtype) showed markedly different CEST profiles. Patient 1 exhibited elevated amide and amine signals within the tumour, while these features were absent in Patient 2. These discrepancies were consistent across field strengths and processing pipelines. Notably, the tumour in Patient 1 was an incidental, asymptomatic finding, whereas Patient 2 presented with neurological symptoms.

### Variability of CEST effects

**Figure 7:**
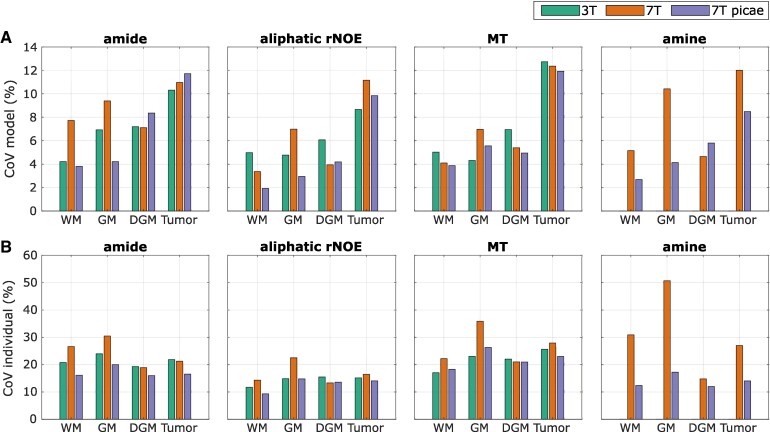
**Variability of CEST metrics across field strengths and processing pipelines.** (**A**) Group-level variability shown as the coefficient of variation (CoV model, %) of model residuals, calculated as the standard deviation of the residuals from the age-dependent fit divided by the mean of the fitted values. (**B**) Individual-level variability (CoV individual, %) defined as the mean within-subject coefficient of variation, calculated as the standard deviation divided by the mean signal within each tissue mask. Bars correspond to results from the three processing pipelines: 3 Tesla (3T; *N* = 36), 7 Tesla (7T, *N* = 32), and 7T with physics-informed conditional autoencoder (PICAE; *N* = 32).

Figure 7 illustrates the variability of CEST quantification across tissue types and processing pipelines. The CoV of the model residuals was elevated in tumour tissue across all pipelines. The CoV at the individual level followed a similar pattern. Across tissues and metrics, conventional 7T data exhibited higher variability than 3T, especially at the individual level. Application of the PICAE pipeline at 7T reduced both the model and individual CoV, achieving values similar or lower than those at 3T, especially for amide and aliphatic rNOE.

## Discussion

CEST imaging has undergone rapid methodological and translational development in neuro-oncology over the past two decades, but its clinical integration remains limited.^37-39^ APTw is currently the most widely implemented variant, although access and deployment are still vendor- and site-dependent rather than universally established.^37,39,40^ Clinically, the most advanced application is the treatment monitoring setting, particularly the differentiation of tumour progression or recurrence from treatment-related change or pseudo progression in glioma, whereas evidence in brain metastases in similar clinical settings remains comparatively limited.^41-44^ Despite these advances, direct comparisons of field strength–dependent sensitivity (3T versus 7T) remain lacking. In this context, this study presents a direct methodological comparison of multi-pool CEST imaging at 3T and 7T in a clinical cohort, with the goal of quantifying how field strength influences the sensitivity and interpretability of CEST contrasts under otherwise comparable conditions.

### Field strength sensitivity to age-related tissue changes

We found that multi-pool CEST at 7T is substantially more sensitive to age-related alterations in normal-appearing brain tissue than at 3T, particularly for the amide and aliphatic rNOE pools in GM and DGM. Age dependence at 7T aligns with previous findings in healthy individuals,^23^ although the average yearly decline in GM for amide and rNOE was approximately twice that previously reported, indicating a steeper age-related signal change. This may reflect microstructural or pH alterations in peritumoural regions, which can influence CEST signals even in tissue that appears normal on conventional imaging.^3,45,46^ Methodological differences may also contribute to this difference: our cohort contained a broader age range (18–75 years) compared to the prior study’s young–elderly comparison, and we used single-transmit set-up, whereas the previous study used parallel transmit coils for improved B_1_ homogeneity. In contrast, age-related effects at 3T were weaker and limited to rNOE (in GM and DGM) and MT (in WM and DGM). Because the biological basis of age-related changes should not differ across field strengths, the reduced sensitivity at 3T likely reflects technical limitations, including lower spectral separation, reduced SNR and diminished T_1_ relaxation effects. These factors may limit the ability of 3T CEST to detect subtle age-related changes.

Age-related changes in T_1_ relaxation times, reported to increase in WM and decrease in GM,^47,48^ may explain the steeper age slopes observed in GM. Although T_1_ mapping was only available for some subjects at 7T, a partial analysis (Supplementary Material 8, Supplementary Fig. 10) reproduced the increase in WM but not the GM decrease. Future studies incorporating T_1_ correction may improve the interpretability and precision of age-sensitive CEST metrics.

Ageing is a key risk factor for neurodegenerative disease,^49,50^ and has been increasingly linked to metabolic alterations.^51-54^ Our findings, together with those of Mennecke *et al*.,^23^ raise the possibility that CEST could serve as a non-invasive imaging marker of biological ageing. Additionally, increasing evidence shows that ageing influences the tumour microenvironment,^55,56^ highlighting the relevance of investigating age-dependent CEST signatures within tumour tissue. In our study, however, tumour compartments demonstrated no age-related trend at either field strength, suggesting age-related effects in tumours are weak compared with subtype variability, reducing the need for age correction in tumour assessments. Our results may serve as reference for the average yearly decline in CEST signal within glioma patients, providing context for interpreting patient-level changes in future longitudinal or comparative studies.

### Field strength sensitivity to tumour-related tissue changes

Although absolute CEST amplitudes did not distinguish IDH mutation status or WHO grade at either field strength, relative contrast to contralateral tissue revealed consistent trends in amide signal at 3T and 7T, in line with prior studies linking amide content to IDH mutation status.^57,58^ However, the lack of statistical significance after correction highlights the influence of biological and technical variability. Notably, differences between tumour types were comparable across field strengths and processing methods. For the proposed acquisition and processing conditions, the improved spectral separation and SNR at 7T did not translate into better subtype differentiation. This is further illustrated by the high inter-patient variability that we observed, including the case of two patients with identical histopathological diagnoses showing divergent CEST profiles but consistent across field strengths. This observation suggests that CEST contrast may be influenced by broader clinical factors beyond molecular subtypes (e.g. tumour growth dynamics, symptom onset, or underlying micro-environmental differences) thereby limiting the robustness of CEST as a biomarker for subtype classification. The apparent variability in CEST responses across patients further motivated our analysis of within-group CoV to assess the reproducibility and stability of CEST metrics across individuals.

### Variability across field strengths

Across most pools and tissues, 7T data showed higher variability than 3T both at the model and individual level, likely reflecting higher sensitivity to B_0_ and B_1_ inhomogeneities under single-transmit conditions. Notably, the variability observed for the 7T Lorentzian fit was higher than previously reported for healthy populations using pTx coils (∼2%).^23^ This highlights that, in a clinical cohort, the technical advantages of 7T can be offset by increased variability when using conventional processing. On the other hand, 7T requires advanced correction to outperform 3T. Applying PICAE substantially reduced variability at 7T, bringing both model- and individual-level CoV to values comparable to, and in most pools lower than, those observed at 3T. This reduction suggests that a large portion of the variability observed at 7T is technical rather than biological in origin and may help reduce the higher rate of dataset exclusion observed at 7T. Therefore, for single-transmit coils, 3T offers more stable CEST measurements under standard processing, whereas 7T can exceed 3T stability only when advanced corrections such as PICAE are applied.

### Study limitations

This study has several limitations. The analysis of age dependence was restricted to normal-appearing tissue in glioma patients, which may limit generalizability to healthy populations. Although we excluded datasets with pronounced artefacts, 7T acquisitions remained vulnerable to B_1_ and B_0_ inhomogeneity, underscoring the importance of optimized acquisition protocols. Additionally, the inability to perform subject-specific T_1_ correction limits our capacity to fully isolate chemical exchange effects from relaxation-driven contrast. Tumour subtyping results may be influenced by the tumour segmentation strategy. A segmentation strategy tailored to the cohort and clinical question should be further explored, as it may influence the specificity of CEST contrasts. While we focused on individual CEST pools to emphasize field strength differences, combining multiple pools in a multi-parametric approach may enhance subtype sensitivity and biological interpretation. Moreover, this study was not powered for tumour subtype discrimination, and all tumour-related findings should be considered exploratory.

## Conclusions

This study provides a direct methodological comparison of multi-pool CEST imaging at 3T and 7T in a clinical cohort. We found that 7T CEST is substantially more sensitive to age-related changes in normal-appearing brain tissue than 3T, particularly in amide and rNOE pools, supporting its potential as a non-invasive biomarker of brain ageing. In contrast, tumour molecular subtypes were not reliably differentiated at either field strength, indicating that current acquisition and processing protocols are insufficient for robust subtype classification. Variability analyses revealed that while 7T offers higher nominal sensitivity than 3T, it also introduces increased technical variability under single-transmit conditions. PICAE quantification substantially reduces this variability, bringing 7T measurements in line with, or below, 3T variability and offering a viable path to enhance the clinical utility of single-transmit 7T CEST. Overall, these results underscore the importance of field strength–specific methodological considerations, including advanced correction strategies, when implementing CEST imaging in clinical settings.

## Supplementary Material

fcag248_Supplementary_Data

## Data Availability

All raw and processed chemical exchange saturation transfer brain imaging data from this study will be openly available upon publication via the University of Bern data repository: https://doi.org/10.48620/96500. The corresponding processing and analysis code is available at: https://github.com/milecap/GliomaCEST-3T7T.git

## References

[1] Halefoğlu AM , ed. High-resolution neuroimaging – basic physical principles and clinical applications. InTech; 2018. 10.5772/intechopen.68268.

[2] Van Zijl PCM, Yadav NN. Chemical exchange saturation transfer (CEST): What is in a name and what isn’t? Magn Reson Med. 2011;65(4):927–948. 21337419 10.1002/mrm.22761 PMC3148076

[3] Jones KM, Pollard AC, Pagel MD. Clinical applications of chemical exchange saturation transfer (CEST) MRI. J Magn Reson Imaging. 2018;47(1):11–27. 28792646 10.1002/jmri.25838 PMC5821273

[4] Wamelink IJHG, Kuijer JPA, Padrela BE, et al Reproducibility of 3 T APT-CEST in healthy volunteers and patients with brain glioma. J Magn Reson Imaging. 2023;57(1):206–215. 35633282 10.1002/jmri.28239 PMC10084114

[5] Wen Z, Hu S, Huang F, et al MR imaging of high-grade brain tumors using endogenous protein and peptide-based contrast. Neuroimage. 2010;51(2):616–622. 20188197 10.1016/j.neuroimage.2010.02.050 PMC2856810

[6] Wu Y, Liu Z, Yang Q, et al Fast and equilibrium CEST imaging of brain tumor patients at 3T. Neuroimage Clin. 2022;33:102890. 34864285 10.1016/j.nicl.2021.102890 PMC8645967

[7] Zou T, Yu H, Jiang C, et al Differentiating the histologic grades of gliomas preoperatively using amide proton transfer-weighted (APTW) and intravoxel incoherent motion MRI. NMR Biomed. 2018;31(1):e3850. 10.1002/nbm.3850 PMC5757627 29098732

[8] Tietze A, Blicher J, Mikkelsen IK, et al Assessment of ischemic penumbra in patients with hyperacute stroke using amide proton transfer (APT) chemical exchange saturation transfer (CEST) MRI. NMR Biomed. 2014;27(2):163–174. 24288260 10.1002/nbm.3048 PMC4019439

[9] Zhang C, Yong XW, Wang Y, et al Effect of saturation pulse power on chemical exchange saturation transfer imaging in patients with acute ischemic stroke. Eur Radiol. 2026;36:1430–1439. 40775093 10.1007/s00330-025-11918-1

[10] Saiyisan A, Zeng S, Zhang H, et al Chemical exchange saturation transfer MRI for neurodegenerative diseases: An update on clinical and preclinical studies. Neural Regen Res. 2026;21(2):553–568. 39885672 10.4103/NRR.NRR-D-24-01246 PMC12220685

[11] Zhang Z, Zhang C, Yao J, et al Protein-based amide proton transfer-weighted MR imaging of amnestic mild cognitive impairment. Neuroimage Clin. 2020;25:102153. 31901792 10.1016/j.nicl.2019.102153 PMC6948365

[12] Zhu D, Fu X, Liu J, et al Multiparametric chemical exchange saturation transfer MRI detects metabolic changes in mild cognitive impairment cases at 3.0 Tesla. Neurochem Res. 2025;50(1):51. 10.1007/s11064-024-04307-5 39648256

[13] Li C, Peng S, Wang R, et al Chemical exchange saturation transfer MR imaging of Parkinson’s disease at 3 Tesla. Eur Radiol. 2014;24(10):2631–2639. 25038850 10.1007/s00330-014-3241-7 PMC4471479

[14] Huang J, Xu J, Lai JHC, et al Relayed nuclear overhauser effect weighted (rNOEw) imaging identifies multiple sclerosis. Neuroimage Clin. 2021;32:102867. 34751151 10.1016/j.nicl.2021.102867 PMC8569719

[15] Dula AN, Asche EM, Landman BA, et al Development of chemical exchange saturation transfer (CEST) at 7T. Magn Reson Med. 2011;66(3):831–838. 21432902 10.1002/mrm.22862 PMC3156337

[16] Vinogradov E, Sherry AD, Lenkinski RE. CEST: From basic principles to applications, challenges and opportunities. Journal of Magnetic Resonance. 2013;229:155–172. 23273841 10.1016/j.jmr.2012.11.024 PMC3602140

[17] Rajput JR, Möhle TA, Fabian MS, et al Physics-informed conditional autoencoder approach for robust metabolic CEST MRI at 7T. In: Greenspan H, et al. eds. Medical Image Computing and Computer Assisted Intervention – MICCAI 2023. MICCAI 2023, Vancouver, BC, Canada. Lecture Notes in Computer Science, vol 14227. Springer, Cham. 10.1007/978-3-031-43993-3_44

[18] Shaffer A, Kwok SS, Naik A, et al Ultra-high-field MRI in the diagnosis and management of gliomas: A systematic review. Front Neurol. 2022;13:857825. 35449515 10.3389/fneur.2022.857825 PMC9016277

[19] Dreher C, Oberhollenzer J, Meissner JE, et al Chemical exchange saturation transfer (CEST) signal intensity at 7T MRI of WHO IV° gliomas is dependent on the anatomic location. J Magn Reson Imaging. 2019;49(3):777–785. 30133046 10.1002/jmri.26215

[20] Schmitz-Abecassis B, de Bresser J, Dirven L, et al Insights into CEST contrast at 2 ppm in enhancing and nonenhancing lesions from glioma patients scanned at 7 T. NMR Biomed. 2025;38(12):e70161. 41102992 10.1002/nbm.70161 PMC12531428

[21] Paech D, Zaiss M, Meissner JE, et al Nuclear overhauser enhancement mediated chemical exchange saturation transfer imaging at 7 Tesla in glioblastoma patients. PLoS One. 2014;9(8):e104181. 25111650 10.1371/journal.pone.0104181 PMC4128651

[22] Yuan Y, Yu Y, Guo Y, et al Noninvasive delineation of glioma infiltration with combined 7T chemical exchange saturation transfer imaging and MR spectroscopy: A diagnostic accuracy study. Metabolites. 2022;12(10):901. 36295803 10.3390/metabo12100901 PMC9607140

[23] Mennecke A, Khakzar KM, German A, et al 7 tricks for 7 T CEST: Improving the reproducibility of multipool evaluation provides insights into the effects of age and the early stages of Parkinson’s disease. NMR Biomed. 2023;36(6):e4717. 35194865 10.1002/nbm.4717

[24] Anemone A, Capozza M, Arena F, et al In vitro and in vivo comparison of MRI chemical exchange saturation transfer (CEST) properties between native glucose and 3-O-methyl-D-glucose in a murine tumor model. NMR Biomed. 2021;34(12):e4602. 34423470 10.1002/nbm.4602 PMC9285575

[25] Lee JS, Xia D, Jerschow A, Regatte RR. In vitro study of endogenous CEST agents at 3T and 7T. Contrast Media Mol Imaging. 2016;11(1):4–14. 26153196 10.1002/cmmi.1652 PMC4706513

[26] Zaiss M, Ehses P, Scheffler K. Snapshot-CEST: Optimizing spiral-centric-reordered gradient echo acquisition for fast and robust 3D CEST MRI at 9.4 T. NMR Biomed. 2018;31(4):e3879. 29372571 10.1002/nbm.3879

[27] Deshmane A, Zaiss M, Lindig T, et al 3D gradient echo snapshot CEST MRI with low power saturation for human studies at 3T. Magn Reson Med. 2019;81:2412–2423. 30431179 10.1002/mrm.27569 PMC6718050

[28] Schuenke P, Windschuh J, Roeloffs V, Ladd ME, Bachert P, Zaiss M. Simultaneous mapping of water shift and B1(WASABI)—Application to field-inhomogeneity correction of CEST MRI data. Magn Reson Med. 2017;77(2):571–580. 26857219 10.1002/mrm.26133

[29] Rebsamen M, Rummel C, Reyes M, Wiest R, McKinley R. Direct cortical thickness estimation using deep learning-based anatomy segmentation and cortex parcellation. Hum Brain Mapp. 2020;41(17):4804–4814. 32786059 10.1002/hbm.25159 PMC7643371

[30] Destrieux C, Fischl B, Dale A, Halgren E. Automatic parcellation of human cortical gyri and sulci using standard anatomical nomenclature. Neuroimage. 2010;53(1):1–15. 20547229 10.1016/j.neuroimage.2010.06.010 PMC2937159

[31] McKinley R, Meier R, Wiest R. Ensembles of densely-connected CNNs with label-uncertainty for brain tumor segmentation. In: Crimi A, Bakas S, Kuijf H, Keyvan F, Reyes M, van Walsum T, eds. Brainlesion: Glioma, Multiple Sclerosis, Stroke and Traumatic Brain Injuries. BrainLes 2018, Granada, Spain. Lecture Notes in Computer Science, Vol. 11384. Springer, Cham. 10.1007/978-3-030-11726-9_40

[32] Zaiß M, Schmitt B, Bachert P. Quantitative separation of CEST effect from magnetization transfer and spillover effects by Lorentzian-line-fit analysis of z-spectra. Journal of Magnetic Resonance. 2011;211(2):149–155. 21641247 10.1016/j.jmr.2011.05.001

[33] Windschuh J, Zaiss M, Meissner JE, et al Correction of B1-inhomogeneities for relaxation-compensated CEST imaging at 7T. NMR Biomed. 2015;28(5):529–537. 25788155 10.1002/nbm.3283

[34] Board WC of TE . Central nervous system tumours. International Agency for Research on Cancer, ed.; 2022. Accessed January 31, 2025. https://www.iarc.who.int/news-events/publication-of-the-who-classification-of-tumours-5th-edition-volume-6-central-nervous-system-tumours/

[35] Kruskal WH, Wallis WA. Use of ranks in one-criterion variance analysis. J Am Stat Assoc. 1952;47(260):583–621.

[36] Gibbons JD, Chakraborti S. Nonparametric statistical inference; 2010. doi:10.1201/9781439896129

[37] Zhou J, Heo HY, Knutsson L, van Zijl PCM, Jiang S. APT-weighted MRI: Techniques, current neuro applications, and challenging issues. J Magn Reson Imaging. 2019;50(2):347–364. 30663162 10.1002/jmri.26645 PMC6625919

[38] Zhou J, Zaiss M, Knutsson L, et al Review and consensus recommendations on clinical APT-weighted imaging approaches at 3T: Application to brain tumors. Magn Reson Med. 2022;88(2):546–574. 35452155 10.1002/mrm.29241 PMC9321891

[39] Nichelli L, Zaiss M, Casagranda S. APT weighted imaging in diffuse gliomas. BJR Open. 2023;5(1):20230025. 37942492 10.1259/bjro.20230025 PMC10630980

[40] Friismose AI, Markovic L, Nguyen N, Gerke O, Schulz MK, Mussmann BR. Amide proton transfer-weighted MRI in the clinical setting—Correlation with dynamic susceptibility contrast perfusion in the post-treatment imaging of adult glioma patients at 3T. Radiography. 2022;28(1):95–101. 34509365 10.1016/j.radi.2021.08.006

[41] Jiang S, Eberhart CG, Lim M, et al Identifying recurrent malignant glioma after treatment using amide proton transfer-weighted MR imaging: A validation study with image-guided stereotactic biopsy. Clin Cancer Res. 2019;25(2):552–561. 30366937 10.1158/1078-0432.CCR-18-1233 PMC6335169

[42] Essed RA, Prysiazhniuk Y, Wamelink IJ, Azizova A, Keil VC. Performance of amide proton transfer imaging to differentiate true progression from therapy-related changes in gliomas and metastases. Eur Radiol. 2024;35(2):580–591. 39134744 10.1007/s00330-024-11004-y PMC11782315

[43] Mehrabian H, Chan RW, Sahgal A, et al Chemical exchange saturation transfer MRI for differentiating radiation necrosis from tumor progression in brain metastasis—Application in a clinical setting. J Magn Reson Imaging. 2023;57(6):1713–1725. 36219521 10.1002/jmri.28440

[44] Ma B, Blakeley JO, Hong X, et al Applying amide proton transfer-weighted MRI to distinguish pseudoprogression from true progression in malignant gliomas. J Magn Reson Imaging. 2016;44(2):456–462. 26788865 10.1002/jmri.25159 PMC4946988

[45] Zaiss M, Windschuh J, Goerke S, et al Downfield-NOE-suppressed amide-CEST-MRI at 7 Tesla provides a unique contrast in human glioblastoma. Magn Reson Med. 2017;77(1):196–208. 26845067 10.1002/mrm.26100

[46] Paech D, Windschuh J, Oberhollenzer J, et al Assessing the predictability of IDH mutation and MGMT methylation status in glioma patients using relaxation-compensated multipool CEST MRI at 7.0 T. Neuro Oncol. 2018;20(12):1661–1671. 29733378 10.1093/neuonc/noy073 PMC6231210

[47] Kupeli A, Kocak M, Goktepeli M, Karavas E, Danisan G. Role of T1 mapping to evaluate brain aging in a healthy population. Clin Imaging. 2020;59(1):56–60. 31760278 10.1016/j.clinimag.2019.09.005

[48] Aktaş Dinçer H, Ağildere AM, Gökçay D. T1 relaxation time is prolonged in healthy aging: A whole brain study. Turk J Med Sci. 2023;53(3):675. 37476907 10.55730/1300-0144.5630 PMC10387954

[49] Baker GT, Sprott RL. Biomarkers of aging. Exp Gerontol. 1988;23(4–5):223–239. 10.1016/0531-5565(88)90025-3 3058488

[50] Moqri M, Herzog C, Poganik JR, et al Validation of biomarkers of aging. Nat Med. 2024;30(2):360–372. 38355974 10.1038/s41591-023-02784-9 PMC11090477

[51] Barzilai N, Huffman DM, Muzumdar RH, Bartke A. The critical role of metabolic pathways in aging. Diabetes. 2012;61(6):1315–1322. 22618766 10.2337/db11-1300 PMC3357299

[52] Finkel T . The metabolic regulation of aging. Nat Med. 2015;21(12):1416–1423. 26646498 10.1038/nm.3998

[53] Houtkooper RH, Argmann C, Houten SM, et al The metabolic footprint of aging in mice. Sci Rep. 2011;1(1):134. 22355651 10.1038/srep00134 PMC3216615

[54] Ma Y, Li J. Metabolic shifts during aging and pathology. Compr Physiol. 2015;5(2):667–686. 25880509 10.1002/cphy.c140041 PMC4968937

[55] Ravensbergen C, van Holstein Y, Hagenaars S, et al Association of biological age with tumor microenvironment in patients with esophageal adenocarcinoma. Gerontology. 2024;70(4):337–350. 38286115 10.1159/000536471 PMC11008718

[56] Zhang C, Neha, Zhang J, et al Aging and senescence: Key players in brain tumor progression and drug resistance. Drug Resist Updat. 2025;81:101228. 40068246 10.1016/j.drup.2025.101228

[57] Jiang S, Wen Z, Ahn SS, et al Applications of chemical exchange saturation transfer magnetic resonance imaging in identifying genetic markers in gliomas. NMR Biomed. 2023;36(6):e4731. 35297117 10.1002/nbm.4731 PMC10557022

[58] Zhang X, Lu J, Liu X, et al Multipool-CEST and CEST-based pH assessment as predictive tools for glioma grading, IDH mutation, 1p/19q codeletion, and MGMT promoter methylation in gliomas. Front Oncol. 2024;14:1507335. 39759149 10.3389/fonc.2024.1507335 PMC11695364

