# Supplementary material for: Field strength–dependent sensitivity of chemical exchange saturation transfer: a methodological comparison of 3 Tesla and 7 Tesla in a clinical cohort
Source: Brain Commun. 2026 Jun 30;8(4):fcag248. doi: 10.1093/braincomms/fcag248 (PMC13348846; doi:10.1093/braincomms/fcag248)
Supplement: fcag248_Supplementary_Data [file fcag248_supplementary_data.docx]

# Supplementary Material

### Supplementary Material 1: Full MRI Acquisition Parameters

*7T acquisition protocol*

CEST imaging at 7T followed a previously described protocol^1^. The pre-saturation module applied two average B_1_ pulse amplitudes (0.72 and 1.00 $\mu T$) using a train of 120 pulses (15.36 ms duration, 10 ms delay), resulting in a ~60% duty cycle. The protocol sampled a total of 54 frequency offsets non-equidistantly between –100 and 100 ppm, with 39 centered between –5 and 5 ppm, and two reference scans at ±300 ppm. Image readout consisted of a 3D snapshot gradient echo (GRE) sequence using centric k-space trajectory ^2^ with: repetition time (TR) = 3.7 ms, echo time (TE) = 1.77 ms, flip angle (FA) = 6°, GRAPPA factor 2 in the first phase encoding direction, field of view (FoV) = 230 x 186 mm, matrix size = 104 x 128 x 18, voxel size = 1.79 x 1.79 x 3 mm^3^ and acquisition time (TA) = 6:42 minutes per B_1_ amplitude. B1 mapping was performed using a Turbo-FLASH sequence (TR = 16 s, TE = 1.68 ms, FoV = 230 × 186 mm², matrix = 104 × 128 × 18, GRAPPA factor 2, TA = 0.33 minutes)^3^.

We additionally acquired high-resolution T2w-FLAIR with the following parameters: TR = 8000 ms, TE = 293 ms, inversion time (TI) = 3120 ms, FA = 120 °, FoV = 189 × 225 mm, matrix size = 270 x 320 x 192, voxel size = 0.7 x 0.7 x 0.7 mm, BW = 650 Hz/pixel, and TA = 06:32 minutes. In 25 subjects, we also acquired MP2RAGE with TR = 6000 ms, TE = 2.06 ms, TI = 800/2700 ms, FA = 4/5 °, FoV = 240 x 240 mm^2^, matrix size = 384 x 384 x 256, voxel size = 0.6 x 0.6 x 0.6 mm^3^, BW = 240 Hz/Px, and TA = 7:40 minutes. For the remaining 18 subjects, we employed an MPRAGE sequence with TR = 2500 ms, TE = 3.12 ms, TI = 1050 ms, FA = 4/5 °, FoV = 225 x 224 mm^2^, matrix size = 374 x 372 x 288, voxel size = 0.6 x 0.6 x 0.6 mm^3^, BW = 235 Hz/Px and TA = 6:51 minutes.

*3T acquisition protocol*

The CEST 3T protocol applied pre-saturation at two average B_1_ pulse amplitudes of 0.6 and 0.9 $\mu T$, using a train of 80 pulses (20.5 ms duration, 10 ms delay), resulting in a ~50% duty cycle. A total of 55 frequency offsets were sampled (non-equidistantly distributed from –100 to 100 ppm, with 45 between –10 and 10 ppm, and one reference scan at –300 Hz), following a previously proposed protocol^4^. Readout used a 3D snapshot GRE sequence (centric k-space)^2^ with: TR = 4 ms, TE = 2 ms, FA = 6°, GRAPPA = 2, FoV = 220 x 180 mm^2^, matrix size = 112 x 94 x 12, voxel size = 1.96 x 1.96 x 5 mm^3^, BW = 797 Hz/Pixel, and TA = 4:16 minutes per B_1_ amplitude. For B_0_ and B_1_ mapping, we used the WASABI sequence with a rectangular pulse (RF amplitude = 3.7 $\mu T$, duration = 5 ms, TA = 2:05 minutes), and the same readout scheme as CEST^5^.

All 3T CEST scans were acquired prior to contrast administration to avoid confounding T1-shortening effects. Additionally, all subjects underwent the acquisition of a FLAIR and MPRAGE before and after gadolinium administration for tumor and tissue segmentation. While the CEST protocol remained consistent across all 3T scans, structural sequences were adjusted to meet clinical needs, resulting in minor variations in acquisition parameters, which are detailed in Supplementary Table 1. For the MPRAGE, the same acquisition was repeated before and after the administration of gadolinium.

| Sequence name | Nsub | TR (ms) | BW (Hz/Px) | TE (ms) | TI (ms) | FA (°) | voxel_size (mm) | matrix_size |
| --- | --- | --- | --- | --- | --- | --- | --- | --- |
| flair_space_sag_iso_1mm | 40 | 5000 | 750 | 388 | 1800 | 120 | 0.5 x 0.5 x 1 | 512 x 512 x 176 |
| flair_tra_3mm | 1 | 8500 | 120 | 93 | 2440 | 150 | 0.4 x 0.4 x 3.3 | 512 x 448 x 42 |
| 3D_Brain_VIEW_FLAIR_SHC_Sag | 1 | 4800 | 899 | 309.4 | 1650 | 90 | 0.5 x 0.5 x 1 | 512 x 512 x 359 |
| 3D_FLAIR_KM_6 | 1 | 8000 | 1067 | 296.1 | 2400 | 90 | 0.8 x 0.8 x 1 | 320 x 320 x 204 |
| t1_mprage_sag_1mm | 36 | 2300 | 200 | 2.26 | 1100 | 8 | 1 x 1 x 1 | 256 x 256 x 176 |
| t1_mpr_adni_sag_iso_1mm_ns | 1 | 1710 | 200 | 2.26 | 1100 | 8 | 1 x 1 x 1 | 256 x 256 x 176 |
| t1_mpr_sag_iso_1mm_ns | 4 | 1800 | 199 | 2.26 | 1100 | 8 | 1 x1 x 1 | 256 x 256 x 176 |
| t1_fl2d_tra | 2 | 331 | 355 | 3.11 | - | 70 | 0.4 x 0.4 x 3.3 | 576 x 524 x 42 |

Supplementary Table 1: Sequence parameters for high-resolution anatomical imaging at 3T. Nsub: Number of subjects. TR: Repetition time. BW: Bandwidth per pixel. TE: Echo time. TI: Inversion time. FA: Flip angle.

### Supplementary Material 2: Inclusion and exclusions in tumor analysis

To calculate the relative difference amplitude between the tumor tissue and the normally appearing tissue in the contralateral side, we only considered subjects where the tumor was fully contained within one hemisphere. Supplementary Figure 1 A) and B) show examples of included subjects, and Supplementary Figure 1 C) and D) show examples of excluded subjects.


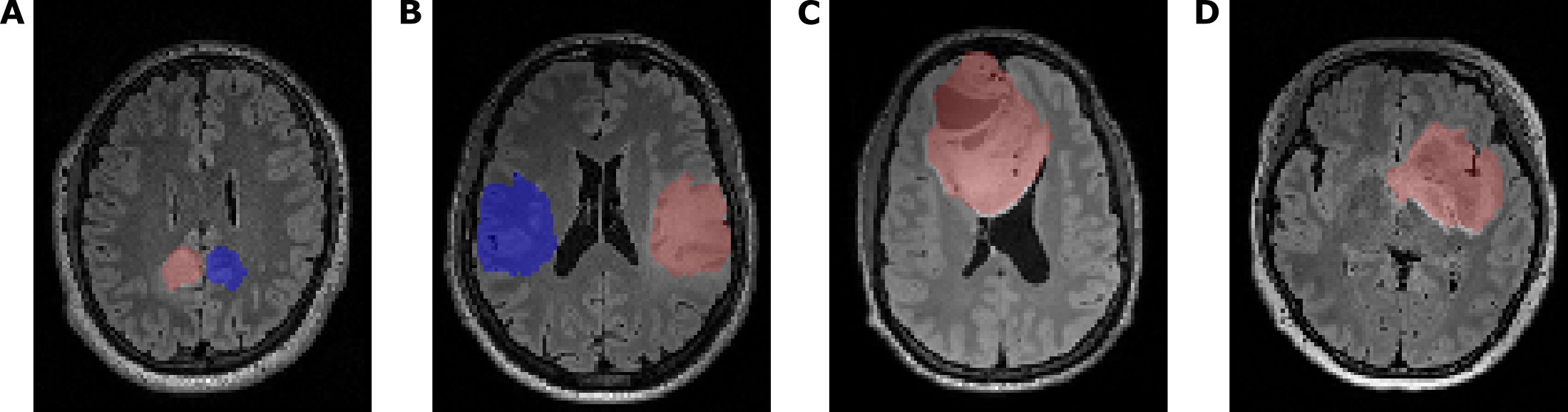


Supplementary Figure 1: Inclusions and Exclusions in Tumor Analysis. A) and B) Examples of subjects included in the tumor analysis. C) and D) Examples of subjects excluded from the analysis due to the inability to identify a contralateral region of normal tissue.

### Supplementary Material 3: Exclusions due to field imperfections

Supplementary Figure 2 a and b shows two subjects with strong B_1_ imperfections excluded from the 7T analysis.


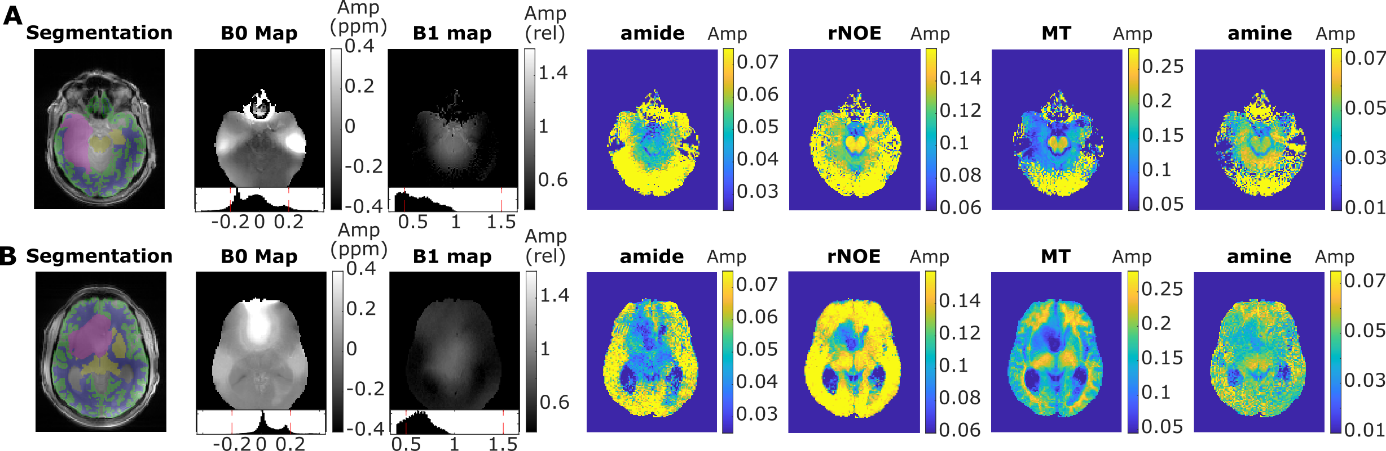


Supplementary Figure 2: Exclusions from the 7T Analysis Due to B1 Field Imperfections. A) Representative example from a single subject (N=1) excluded due to strong B1 field imperfections. B) Representative example from a single subject (N=1) excluded from both the 3T and 7T analysis due to mis segmentation caused by enlarged ventricles. The colormap and corresponding color scale represent the amplitude (Amp) of the Lorentzian fitting for each CEST pool in dimensionless units.

Supplementary Figure 3 shows the age distribution of the initially enrolled subjects, as well as the age distributions of the included subjects at 3T and 7T.


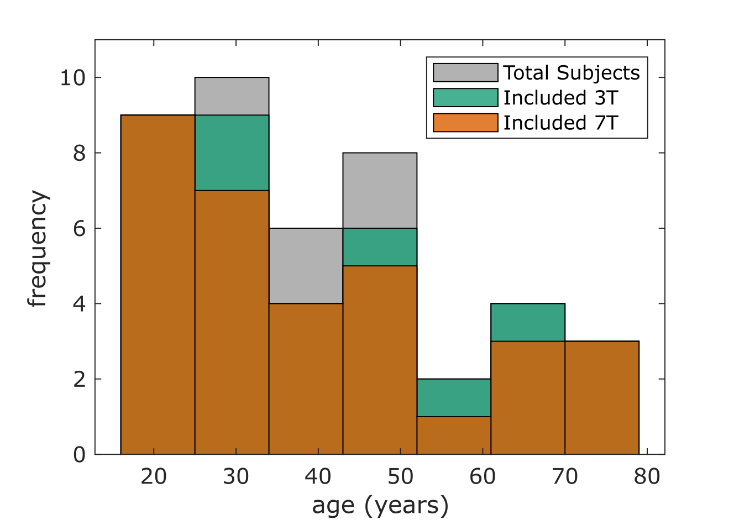


Supplementary Figure 3: Age distribution of all enrolled subjects (N = 43, grey), and final subjects included at 3T (N = 36, green) and 7T (N = 32, orange).

### Supplementary Material 4: B₀ and B₁ field maps at 3T and 7T for two representative patients

Supplementary Figure 4 shows the B₀ and B₁ field maps for the two patients presented in Figure 3 of the main text, acquired at both 3T and 7T. These maps are provided for reference to illustrate the field inhomogeneity characteristics underlying the CEST quantification shown in Figure 3.


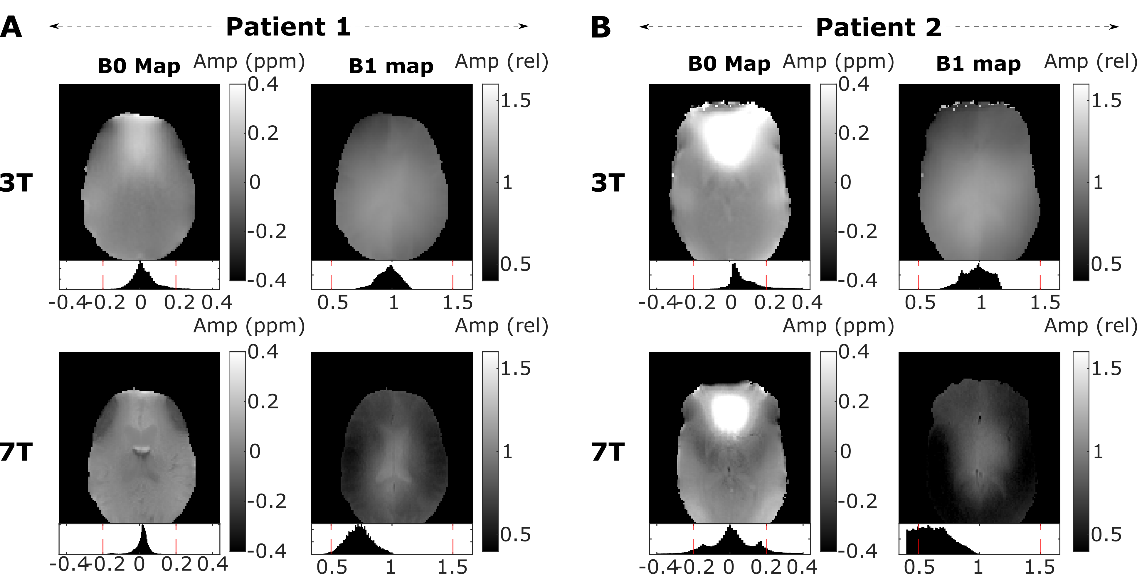


Supplementary Figure 4: B₀ (ppm) and B₁ (relative) field maps for the two subjects shown in Figure 3 of the main text, acquired at 3T and 7T.

### Supplementary Material 5: Additional examples with standard clinical images.

Supplementary Figures 5–8 present examples of CEST contrast maps alongside standard clinical MRI sequences (3D FLAIR, MPRAGE, and post-contrast MPRAGE). For each subject, high-resolution anatomical images and corresponding CEST maps are shown in their native acquisition space (i.e., without cross-registration), with slices selected to match as closely as possible across modalities. The cases shown span from low grade to high grade and smallest to largest in size, highlighting the heterogeneity of our cohort.


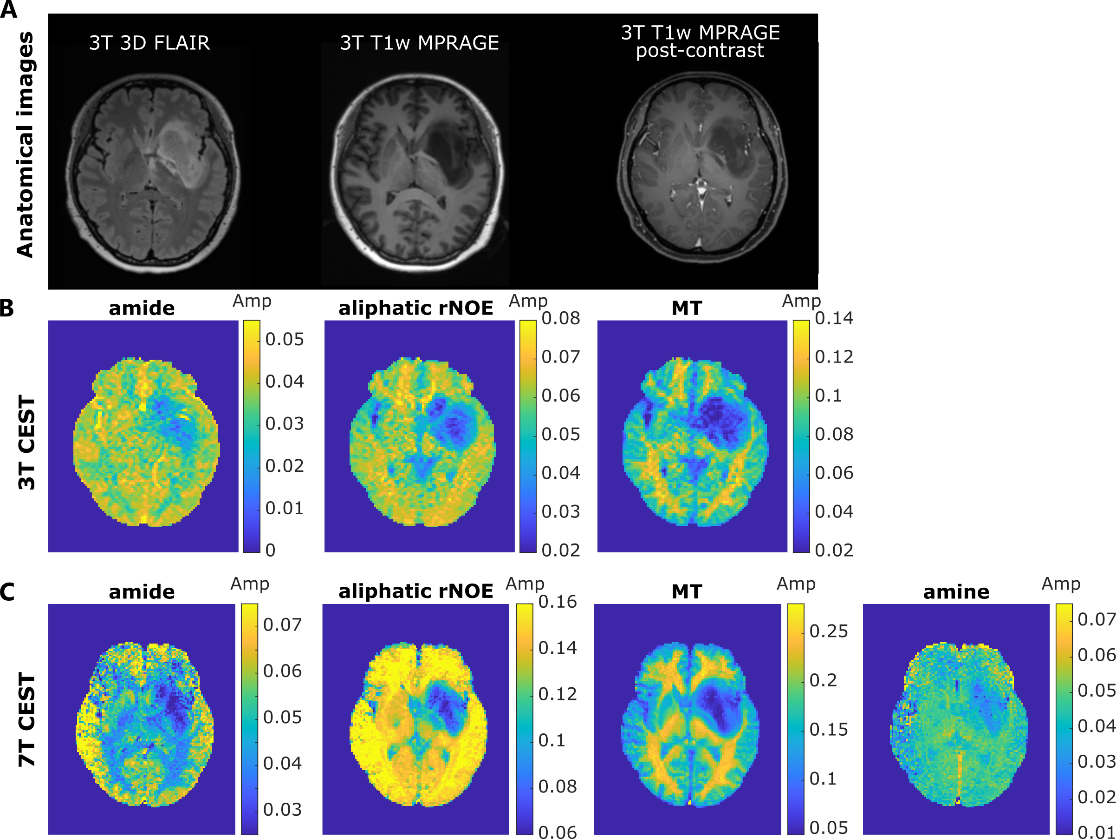


Supplementary Figure 5: Example of a 46-year-old female patient with WHO grade 2, IDH-mutated astrocytoma. A) anatomical MRI acquisitions (T2-weighted FLAIR, MPRAGE, and post-contrast MPRAGE). B) 3T CEST contrast maps for the amide, aliphatic rNOE, and MT pools. C) 7T CEST contrast maps for the amide, aliphatic rNOE, MT, and amine pools. Color scales represent the fitted Lorentzian amplitude (Amp) of each CEST pool in arbitrary units (a.u.).


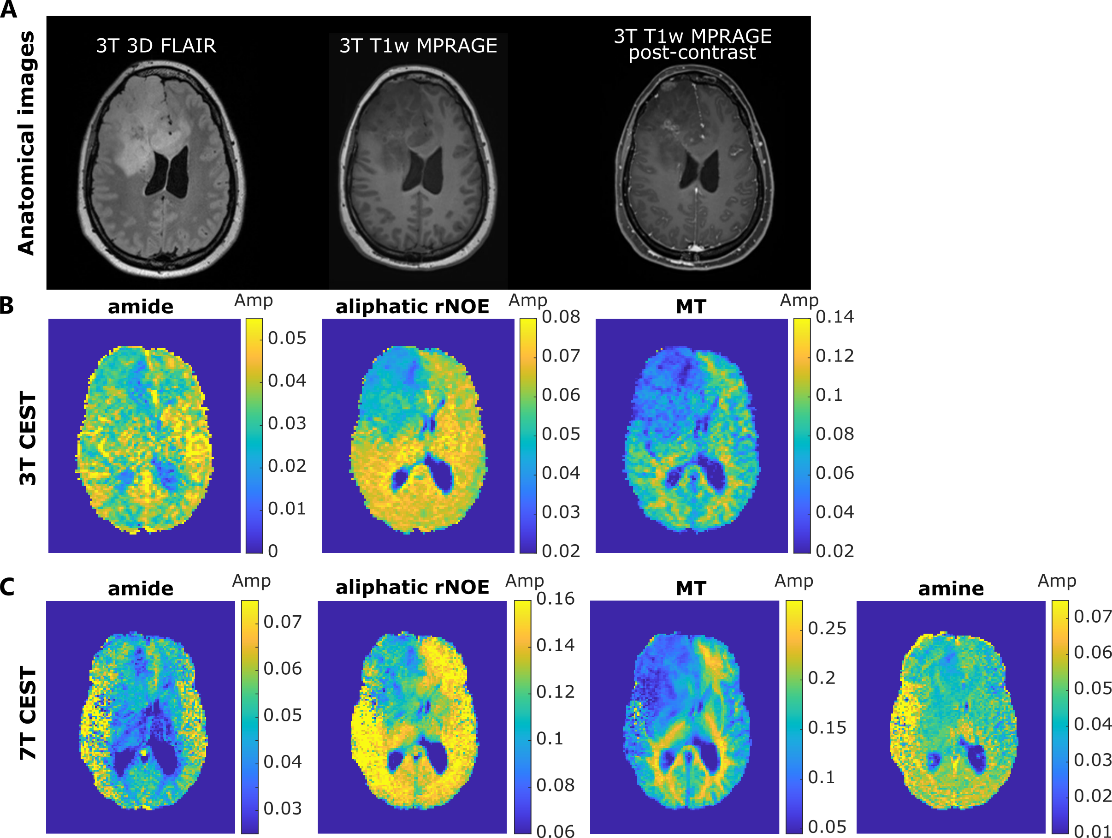


Supplementary Figure 6: Example of a 34-year-old female patient with WHO grade 3, IDH-mutated,1p/19q co-deleted Oligodendroglioma. A) anatomical MRI acquisitions (T2-weighted FLAIR, MPRAGE, and post-contrast MPRAGE). B) 3T CEST contrast maps for the amide, aliphatic rNOE, and MT pools. C) 7T CEST contrast maps for the amide, aliphatic rNOE, MT, and amine pools. Color scales represent the fitted Lorentzian amplitude of each CEST pool in arbitrary units (a.u.).


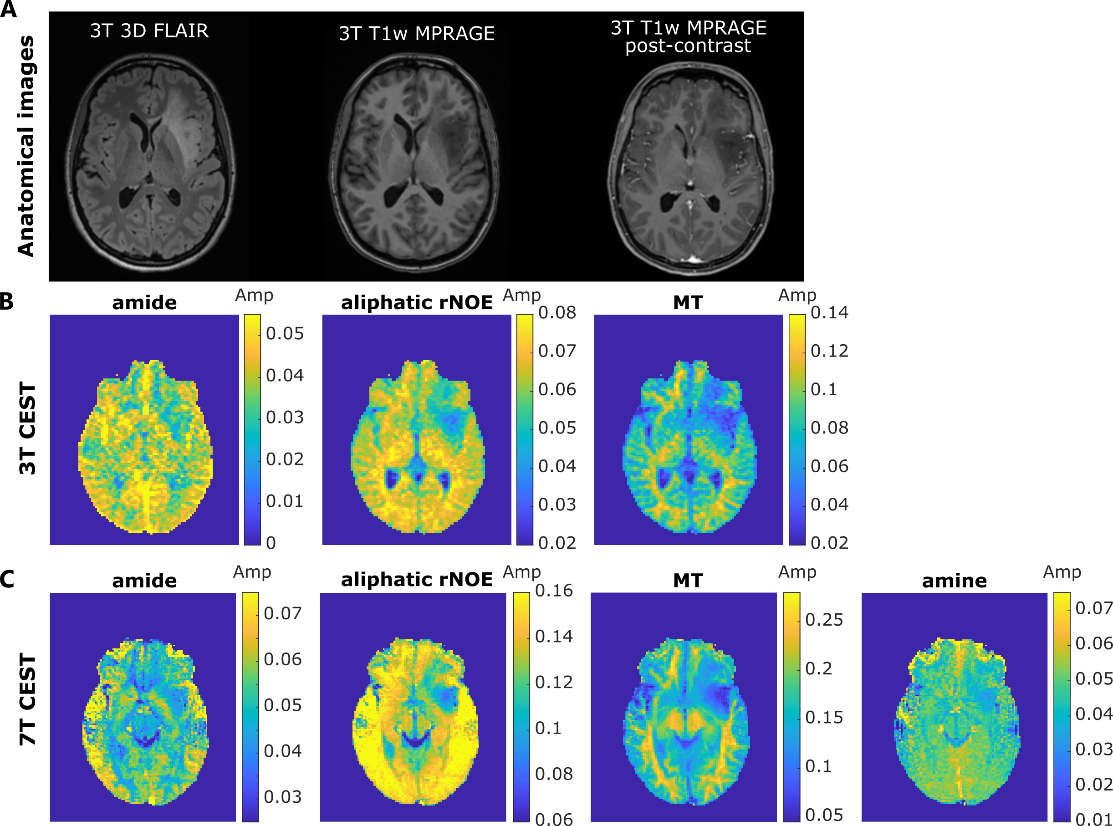


Supplementary Figure 7: Example of a 30-year-old female patient with WHO grade 2, IDH-mutated, 1p/19q co-deleted Oligodendroglioma. A) anatomical MRI acquisitions (T2-weighted FLAIR, MPRAGE, and post-contrast MPRAGE). B) displays 3T CEST contrast maps for the amide, aliphatic rNOE, and MT pools. C) 7T CEST contrast maps for the amide, aliphatic rNOE, MT, and amine pools. Color scales represent the fitted Lorentzian amplitude of each CEST pool in arbitrary units (a.u.).


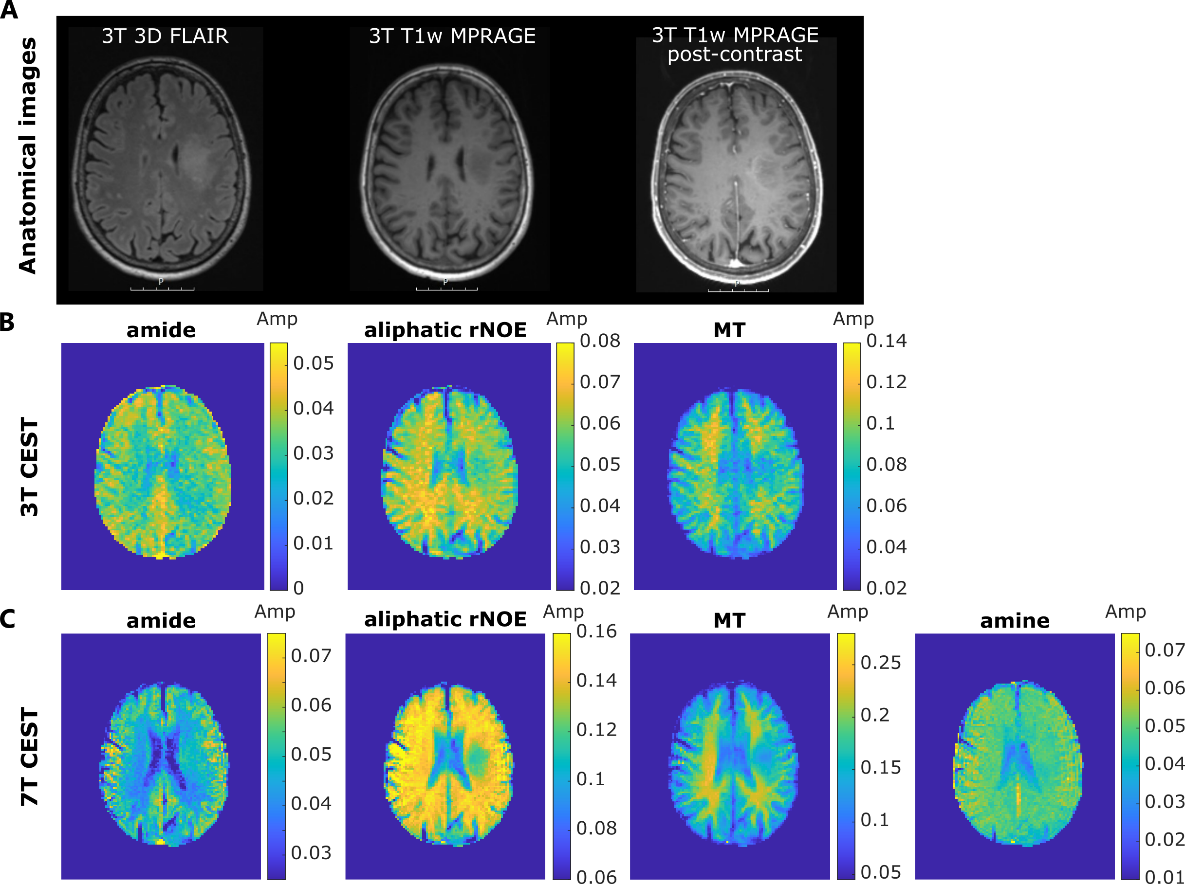


Supplementary Figure 8: Example of a 71-year-old female patient with WHO grade 4, IDH-wildtype, Glioblastoma. A) anatomical MRI acquisitions (T2-weighted FLAIR, MPRAGE, and post-contrast MPRAGE). B) 3T CEST contrast maps for the amide, aliphatic rNOE, and MT pools. C) 7T CEST contrast maps for the amide, aliphatic rNOE, MT, and amine pools. Color scales represent the fitted Lorentzian amplitude of each CEST pool in arbitrary units (a.u.).

### Supplementary Material 6: Summary table of statistical tests

Supplementary Tables 2 and 3 show the regression outcomes of the linear regression analysis when considering sex (in all tissues) and tumor type (only in tumor tissue) covariates.

| Tissue | Pool | R^2^ | C_age_  - 95%CI | Average (%) | p_age_ | Significant |
| --- | --- | --- | --- | --- | --- | --- |
| WM | Amide | 0.2108 | -0.00010 (-0.00020, -0.00000) | 0.2149 | 0.0096 | False |
| WM | rNOE | 0.2136 | -0.00020 (-0.00030, -0.00000) | 0.1 | 0.0095 | False |
| WM | MT | 0.2312 | -0.00030 (-0.00040, -0.00010) | 0.1268 | 0.0063 | False |
| WM | Amine | 0.1686 | -0.00010 (-0.00010, 0.00000) | 0.101 | 0.0645 | False |
| GM | Amide | 0.4398 | -0.00030 (-0.00040, -0.00010) | 0.4043 | 0.0001 | True |
| GM | rNOE | 0.284 | -0.00040 (-0.00060, -0.00010) | 0.2285 | 0.0021 | True |
| GM | MT | 0.2489 | -0.00030 (-0.00060, -0.00010) | 0.2198 | 0.0043 | True |
| GM | Amine | 0.2333 | -0.00020 (-0.00030, -0.00000) | 0.2774 | 0.0097 | False |
| DGM | Amide | 0.2042 | -0.00010 (-0.00020, -0.00000) | 0.1866 | 0.0138 | False |
| DGM | rNOE | 0.3861 | -0.00020 (-0.00030, -0.00010) | 0.1675 | 0.0002 | True |
| DGM | MT | 0.3143 | -0.00040 (-0.00060, -0.00020) | 0.1934 | 0.0013 | True |
| DGM | Amine | 0.27 | -0.00010 (-0.00010, -0.00000) | 0.1551 | 0.0028 | True |
| Tumor | Amide | 0.0278 | -0.00010 (-0.00020, 0.00010) | 0.1023 | 0.3798 | False |
| Tumor | rNOE | 0.0685 | -0.00020 (-0.00050, 0.00010) | 0.1333 | 0.2333 | False |
| Tumor | MT | 0.2317 | -0.00040 (-0.00070, -0.00000) | 0.2396 | 0.0403 | False |
| Tumor | Amine | 0.0268 | 0.00000 (-0.00010, 0.00010) | 0.0307 | 0.8115 | False |

Supplementary Table 2: Linear regression results for each tissue and CEST pool combination at 7T. Shown are the R² values, age coefficients (C_age_) with 95% confidence intervals (lower, upper), uncorrected p-values for age p_age_, and significance after Holm-Bonferroni correction. These results correspond to the age-related CEST effects shown in Figure 4.

| Tissue | Pool | R^2^ | C_age_  - 95%CI | Average (%) | p_age_ | Significant |
| --- | --- | --- | --- | --- | --- | --- |
| WM | Amide | 0.0064 | 0.00000 (-0.00000, 0.00000) | 0.0032 | 0.94236 | False |
| WM | rNOE | 0.0949 | -0.00010 (-0.00010, 0.00000) | 0.0869 | 0.09294 | False |
| WM | MT | 0.2332 | -0.00020 (-0.00030, -0.00010) | 0.1494 | 0.00353 | True |
| GM | Amide | 0.0646 | -0.00000 (-0.00010, 0.00000) | 0.0931 | 0.19549 | False |
| GM | rNOE | 0.2771 | -0.00010 (-0.00010, -0.00000) | 0.1457 | 0.00259 | True |
| GM | MT | 0.2119 | -0.00010 (-0.00020, -0.00000) | 0.1237 | 0.00664 | False |
| DGM | Amide | 0.1203 | -0.00000 (-0.00010, 0.00000) | 0.0915 | 0.19293 | False |
| DGM | rNOE | 0.2758 | -0.00010 (-0.00020, -0.00000) | 0.1484 | 0.00536 | True |
| DGM | MT | 0.3527 | -0.00020 (-0.00030, -0.00010) | 0.2205 | 0.00023 | True |
| Tumor | Amide | 0.0137 | 0.00000 (-0.00010, 0.00010) | 0.0012 | 0.99165 | False |
| Tumor | rNOE | 0.0975 | -0.00010 (-0.00020, 0.00000) | 0.1662 | 0.08486 | False |
| Tumor | MT | 0.0211 | -0.00010 (-0.00030, 0.00010) | 0.1181 | 0.43882 | False |

Supplementary Table 3: Linear regression results for each tissue and CEST pool combination at 3T. Shown are the R² values, age coefficients (C_age_) with 95% confidence intervals (lower, upper), uncorrected p-values for age p_age_, and significance after Holm-Bonferroni correction. These results correspond to the age-related CEST effects shown in Figure 5.

### Supplementary Material 7:

Supplementary Figure 9 shows the comparison of mean CEST pool amplitudes across WHO grade tumors (1 to 4). No significant differences are present among the groups after Holm-Bonferroni correction at α = 0.05.


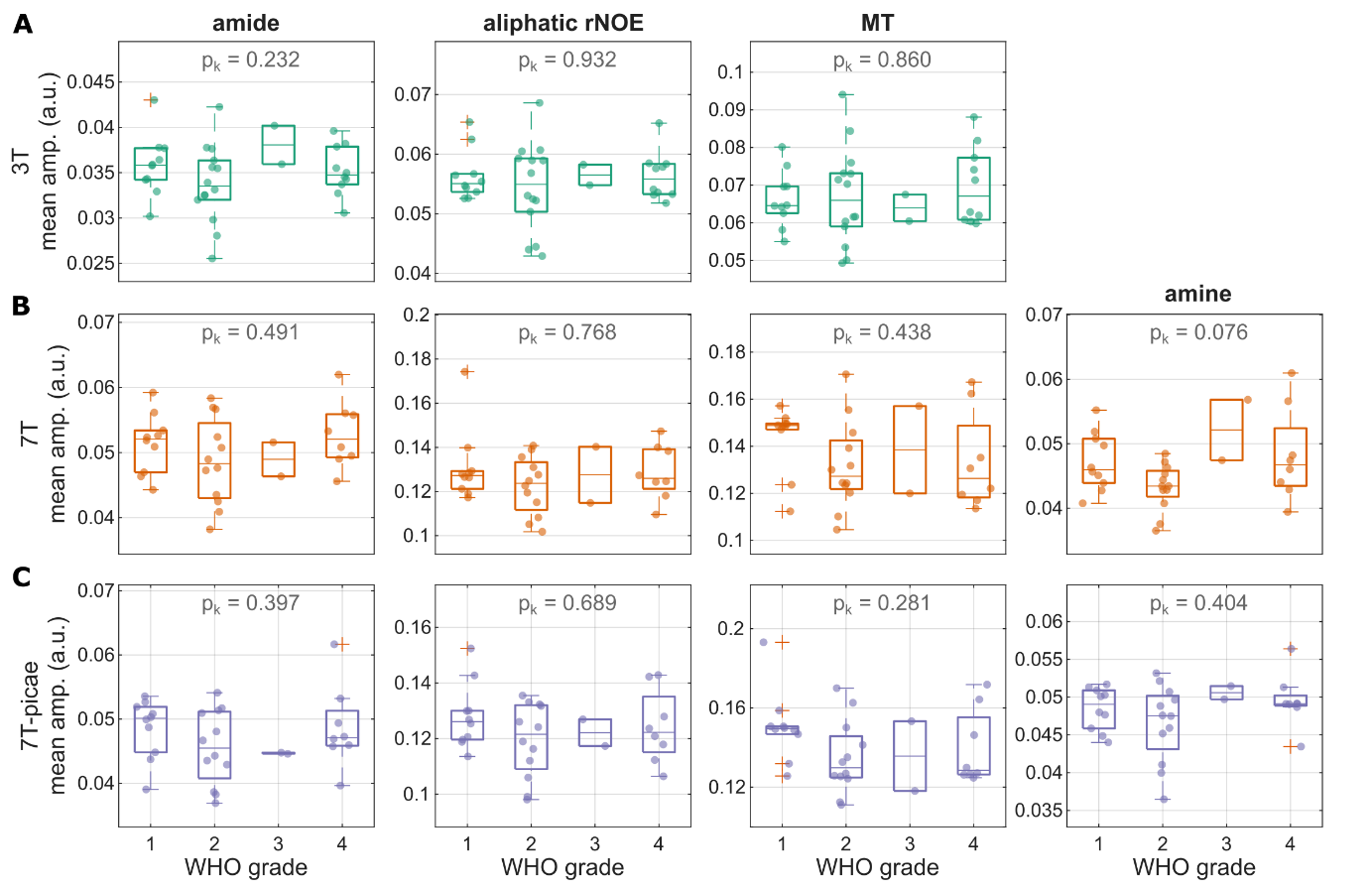


Supplementary Figure 9: Comparison of CEST pool amplitudes across histopathological confirmed WHO grades. a) Boxplots show the mean tumor amplitudes for each CEST pool (amide, aliphatic rNOE, MT, and amine) across WHO grades (1 to 4) at A) 3T (N = 36, green, N_1_=10, N_2_=14, N_3_=2, N_4_=10), B) 7T (N = 32, orange, N_1_=10, N_2_=12, N_3_=2, N_4_=8), and C) 7T PICAE (N = 32, purple, N_1_=10, N_2_=12, N_3_=2, N_4_=8), individual datapoints representing the mean tumor signal for each patient are overlaid. p-values are from two-sided Kruskal–Wallis tests; significance was evaluated at α = 0.05 using Holm–Bonferroni correction, and uncorrected p-values are shown above each comparison.

### Supplementary Material 8:

Supplementary Figure 10 shows T_1_ values as a function of age for each tissue type from the 25 subjects with available MP2RAGE acquisition at 7T. The observed T_1_ increase in WM suggests stronger age-related trends for CEST pools in this tissue, consistent with previous findings^12^. For GM, we did not observe a significant trend, though the decreasing T1 trend reported in the literature might explain the stronger age effects seen in GM. Further work with comprehensive T1 mapping is required to clarify these findings.


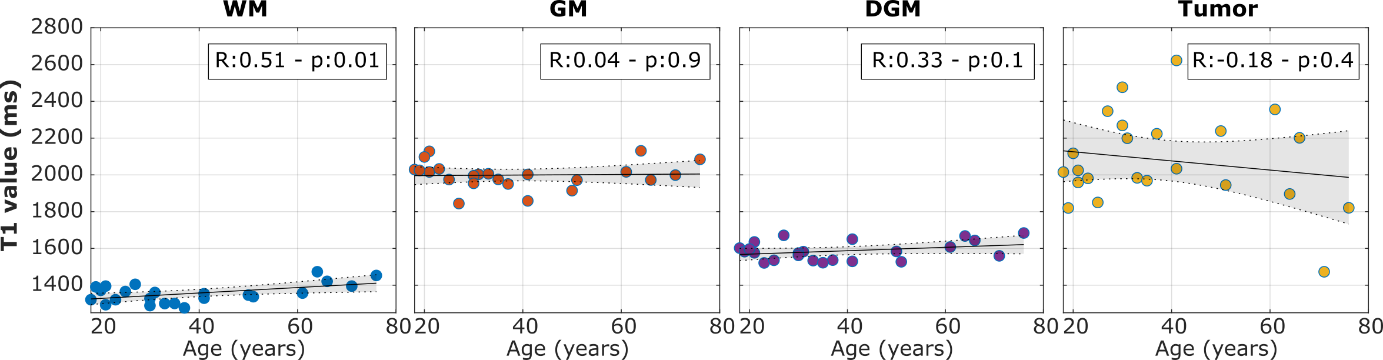


Supplementary Figure 10: Age dependence of T1 values across tissue types at 7T. Age-related changes in T1 values estimated from the MP2RAGE sequence across white matter (WM), grey matter (GM), deep grey matter (DGM), and tumor. Each point represents the mean value for one patient (N = 25). Each plot shows the Pearson correlation coefficient (R) and significance (p-value), with the 95% confidence interval of the linear fit displayed in shaded grey area.

# Supplementary references

1. Mennecke A, Khakzar KM, German A, et al. 7 tricks for 7 T CEST: Improving the reproducibility of multipool evaluation provides insights into the effects of age and the early stages of Parkinson’s disease. *NMR Biomed*. 2023;36(6):e4717. doi:10.1002/NBM.4717

2. Zaiss M, Ehses P, Scheffler K. Snapshot-CEST: Optimizing spiral-centric-reordered gradient echo acquisition for fast and robust 3D CEST MRI at 9.4 T. *NMR Biomed*. 2018;31(4):e3879. doi:10.1002/NBM.3879

3. Windschuh J, Zaiss M, Meissner JE, et al. Correction of B1-inhomogeneities for relaxation-compensated CEST imaging at 7T. *NMR Biomed*. 2015;28(5):529-537. doi:10.1002/NBM.3283

4. Deshmane A, Moritz Zaiss |, Lindig T, et al. 3D gradient echo snapshot CEST MRI with low power saturation for human studies at 3T. Published online 2018. doi:10.1002/mrm.27569

5. Schuenke P, Windschuh J, Roeloffs V, Ladd ME, Bachert P, Zaiss M. Simultaneous mapping of water shift and B1(WASABI)—Application to field-Inhomogeneity correction of CEST MRI data. *Magn Reson Med*. 2017;77(2):571-580. doi:10.1002/MRM.26133/ASSET/SUPINFO/MRM26133-SUP-0001-SUPPINFO.DOCX
